# A model-driven framework for data-driven applications in serverless cloud computing

**DOI:** 10.1371/journal.pone.0237317

**Published:** 2020-08-28

**Authors:** Fatima Samea, Farooque Azam, Muhammad Rashid, Muhammad Waseem Anwar, Wasi Haider Butt, Abdul Wahab Muzaffar

**Affiliations:** 1 Department of Computer and Software Engineering, National University of Sciences and Technology (NUST), Islamabad, Pakistan; 2 Computer Engineering Department, College of Computer and Information Systems, Umm Al-Qura University, Makkah, Saudi Arabia; 3 College of Computing and Informatics, Saudi Electronic University, Riyadh, Saudi Arabia; Lingnan University, HONG KONG

## Abstract

In a serverless cloud computing environment, the cloud provider dynamically manages the allocation of resources whereas the developers purely focus on their applications. The data-driven applications in serverless cloud computing mainly address the web as well as other distributed scenarios, and therefore, it is essential to offer a consistent user experience across different connection types. In order to address the issues of data-driven application in a real-time distributed environment, the use of GraphQL (Graph Query Language) is getting more and more popularity in state-of-the-art cloud computing approaches. However, the existing solutions target the low level implementation of GraphQL, for the development of a complex data-driven application, which may lead to several errors and involve a significant amount of development efforts due to various users’ requirements in real-time. Therefore, it is critical to simplify the development process of data-driven applications in a serverless cloud computing environment. Consequently, this research introduces UMLPDA (**U**nified **M**odeling **L**anguage **P**rofile for **D**ata-driven **A**pplications), which adopts the concepts of UML-based Model-driven Architectures to model the frontend as well as the backend requirements for data-driven applications developed at a higher abstraction level. Particularly, a modeling approach is proposed to resolve the development complexities such as data communication and synchronization. Subsequently, a complete open source transformation engine is developed using a Model-to-Text approach to automatically generate the frontend as well as backend low level implementations of Angular2 and GraphQL respectively. The validation of proposed work is performed with three different case studies, deployed on Amazon Web Services platform. The results show that the proposed framework enables to develop the data-driven applications with simplicity.

## Introduction

The serverless cloud computing allows the user to build and deploy applications without managing the complex infrastructure [1]. Amazon was the first one to introduce a serverless paradigm in year 2014 by introducing Lambda [2]. Nowadays, almost every cloud provider (e.g., Google [3] and Microsoft [4]) offers a serverless platform. As compared to traditional approaches, the applications developed using serverless cloud computing (also known as serverless architectures) either depend on the third-party services (known as Backend-as-a-Service) or on the custom code runs in some stateless compute containers (known as Function-as-a-Service) which are short-lived, event-triggered and fully managed by the cloud provider [1–4]. It implies that the developers write concise and stateless functions which can be triggered through the events, produced from different sensors as well as services / users or middleware [5, 6]. Consequently, the platform ensures a secure and timely execution of these functions (written by developers) by running the infrastructure efficiently [7]. Furthermore, the handling of client requests, tasks scheduling, and capacity planning are performed by the cloud provider.

Several applications have been developed using serverless architectures. However, the data-driven applications, requiring tedious and error prone development efforts, are frequently used on multiple devices (e.g. mobiles, computers, and smartphones) [8]. The core of data-driven applications is data visualization and manipulation such that their behavior is dependent on the input data. These applications are delivered through the web, where the server-side code runs at a cloud while the client application is accessed through a web browser [9, 10]. Generally speaking, the most modern applications take the benefit of RESTful web services for accessing the data that uses HTTP (Hyper Text Transfer Protocol) requests to GET, PUT, POST and DELETE the data [11]. In the context of data-driven applications using REST (Representational State Transfer) for a serverless backend, a set of Lambda functions behind an API (Application Programming Interface) gateway are used to access the data. However, today’s web and mobile applications are data-driven and require larger sets of data which are distributed across the devices and users. Therefore, accessing the data using REST API offers limited query capabilities and developers are required to build some ad-hoc solutions for complex data queries [12].

The industrial solutions for the development of data-driven applications in a serverless cloud computing environment are based on various libraries and frameworks [1, 13]. For example, GraphQL (Graph Query language) [14] is one of the most popular data query language which has been frequently employed. However, the development of a complete data-driven application, using the low level implementation of GraphQL, is a tedious and error prone task due to changing users’ requirements, specifically for the web user interface and interactivity [9, 10]. Particularly, the synchronization of data with the user interface is difficult when the real-time data is distributed among multiple users with different device connection types. Moreover, the integration of application logic with the backend GraphQL API, for data communication and synchronization, is a challenging task. Furthermore, the low-level implementation complexity increases with respect to the behavior of application.

The current state-of-the-art approaches (details are provided in Table 1) do not provide an appropriate modeling solution at higher abstraction level along with the complete tool support for simplifying the behavioral aspects of data-driven applications based on GraphQL in serverless cloud computing. Therefore, it is essential to provide a framework that can simplify the overall development process. Consequently, the proposed work in this article is the first attempt towards the data-driven applications development in a serverless cloud computing environment through a model-driven approach.

**Table 1:** Comparison of state-of-the-art approaches with the proposed work.

Ref. No	Serverless Computing	Data-driven Applications	Frontend Prototype	Backend	Validation on Cloud Providers	Modeling Approach	Tool Support
[9, 31, 33]	No	Yes	Yes	Yes	No	No	No
[28, 29]	No	Yes	No	Yes	No	No	No
[32]	No	Yes	No	Yes	No	No	No
[34, 35, 36, 37]	No	Yes	No	Yes	Yes	Yes	Yes
[38, 39, 42, 43]	Yes	No	No	Yes	Yes	No	No
[41]	Yes	Yes	No	Yes	Yes	No	No
[44]	Yes	No	No	Yes	Yes	Yes	Yes
[45]	Yes	No	Yes	Yes	No	No	No
**This Work**	**Yes**	**Yes**	**Yes**	**Yes**	**Yes**	**Yes**	**Yes**

Model-driven architecture (MDA) provides an abstract representation of software engineering methods, using models as prime artifacts [15]. The functionality of a system is defined without having any technology-specific implementation information i.e. as a platform-independent model [16]. In the context of data-driven applications, the MDA approach must provide the support for modeling frontend, backend and behavioral concepts at platform independent level with the real-time data updates in a serverless cloud computing environment [12]. Subsequently, some appropriate transformation techniques convert the platform-independent models into platform-specific models [17, 18]. The MDA model is related to multiple standards. Among them, the UML (Unified Modeling Language) is one of the most powerful language and has been used by many software engineers for various real-life applications [15–18].

Although the UML provides a standard method to model the structural and behavioral characteristics of a system. However, due to diverse application domains, some additional features may be required by the developers which are not already defined in the UML standard. In other words, developers may require to incorporate some non-semantic information into their application models. Therefore, in order to handle the requirements of diverse application domains, the UML provides a built-in extension mechanism which enables the developers to customize their models for some specific platforms and application domains. Consequently, this article extends the UML diagrams for data-driven applications.

**Fig 1:**
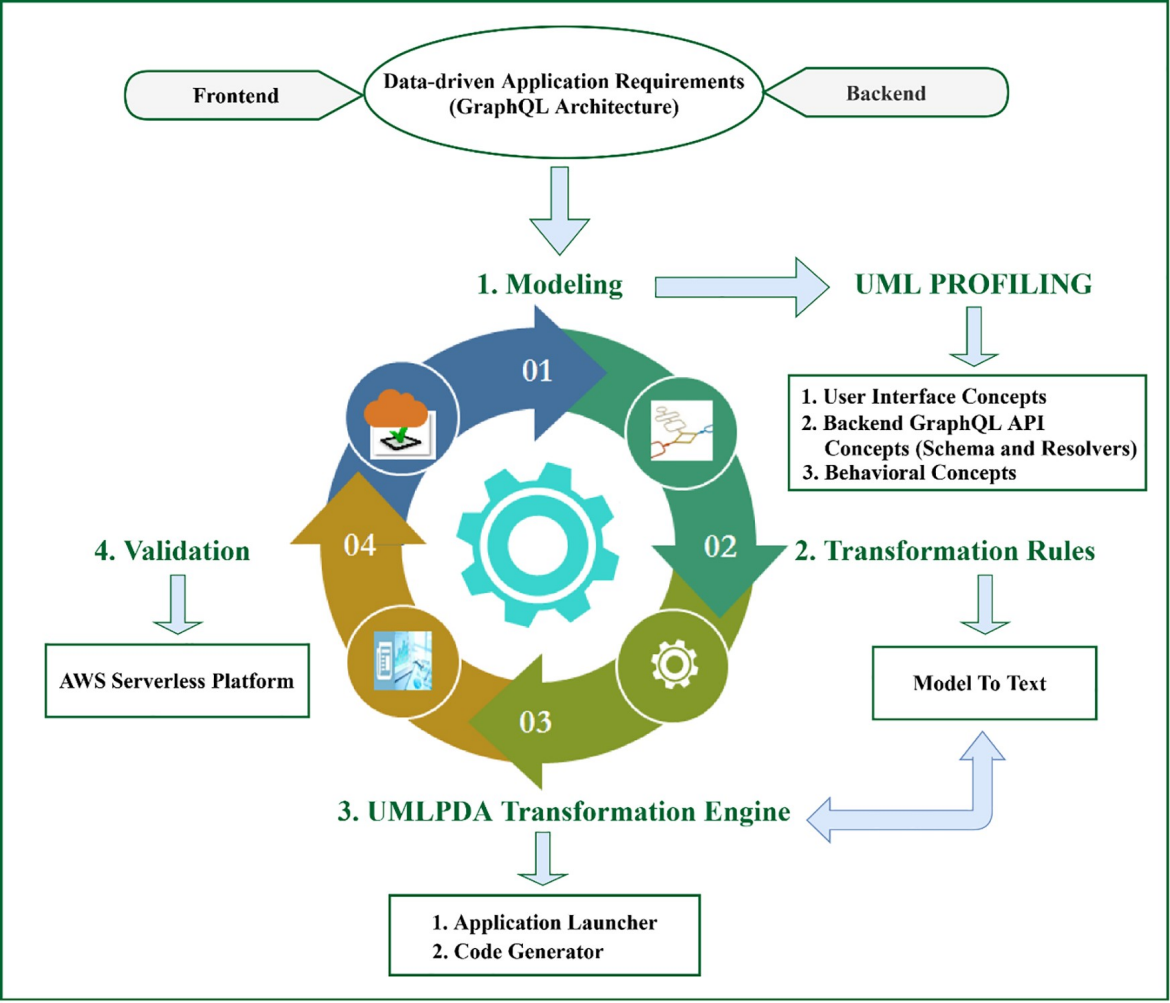
Proposed model-driven framework for data-driven applications using UMLPDA.

In this research, we introduce UMLPDA (**U**nified **M**odeling **L**anguage **P**rofile for **D**ata-driven **A**pplications) to represent the modeling of data-driven applications for both frontend and backend in serverless cloud computing at a higher abstraction level. The salient features of the proposed profile (and hence the research contributions of this article) are shown in Fig 1 and summarized as follows:

1. The frontend of data-driven applications is modelled at platform independent level for the user interface (UI) design and development.
2. The modeling of behavioral concepts is performed to create a client that communicates with the backend cloud data sources and binds the corresponding data to the UI.
3. The modeling of backend GraphQL API concepts is carried out to create GraphQL schema and GraphQL resolvers.
4. An open source transformation engine, which mainly consists of application launcher and code generator, is developed using a Model-to-text (M2T) approach to automatically generate the frontend as well as backend low-level implementations of Angular2 [19] and GraphQL [14]. For frontend, Angular2 has been targeted which has been developed by Google and widely used in the development of web-based user-friendly applications for desktop and mobile devices [20]. For backend, GraphQL [14] has been targeted which is a data query language for the web-based APIs and is most popular nowadays.

The work in this article is significant to the existing body of research knowledge in two different ways: (a) From an application developer’s perspective, the proposed work provides two major benefits: simplicity and reusability [21, 22]. The simplicity is achieved by providing a model-based solution at higher abstraction level. Instead of writing the data-driven applications code at lower abstraction level (which is complex, time-consuming and error-prone), the low level application code is generated automatically through the proposed transformation engine which ultimately reduces the development complexities and time. The reusability of the proposed solution lies in the fact that the platform independent model can be reused to implement the same system for different target technologies. For example, currently we have targeted Angular2 and GraphQL. However, the proposed high level models can be reused to generate the low level implementations of React.js, Vue.js etc. with GraphQL. (b) From an end user’s perspective, the application provides an instant access to the data, both offline as well as online, along with the real-time data updates, under different device connection types.

The validation of the proposed UMLPDA profile is performed with three case studies by deploying it on the Amazon Web Services (AWS) platform. We have selected the AWS as it is among the top competitors in the cloud market and most leading in academia [23]. The paper is organized as follows: First, the preliminary concepts and state-of-the-art approaches are discussed. Then the proposed profile and its transformation engine are described for data-driven applications in a serverless cloud computing environment. Subsequently, the validation of the proposed approach is presented by applying it on three different case studies. Finally, an appropriate conclusion is provided.

## Preliminaries

It has been stated in the introductory part of this article that there are two types of serverless architectures: FaaS (Functions-as-a-Service) and BaaS (Backend-as-a-Service). In FaaS, the application developer writes the custom logic which is executed in some stateless containers while the BaaS configuration allows the developer to create powerful applications by linking the available services from various cloud providers [24]. This section highlights the background of data-driven applications architecture in serverless cloud computing. Furthermore, it also discusses state-of-the-art approaches and research gaps.

### Data-driven applications architecture in a serverless cloud

#### Computing environment

**Fig 2:**
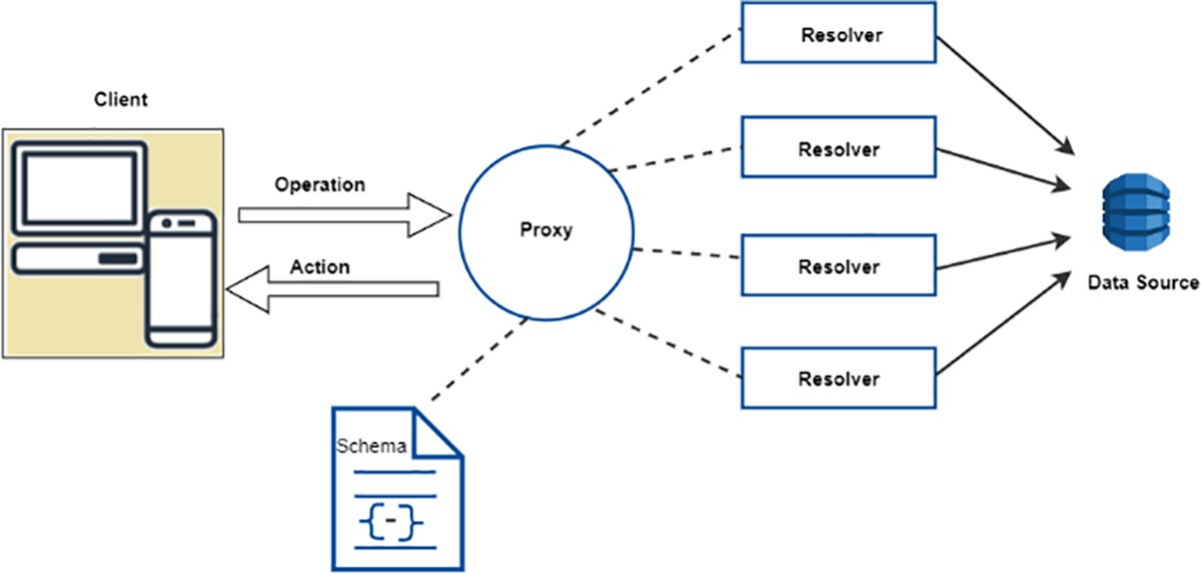
The architecture for data-driven applications in a serverless cloud computing.

In this fast-paced modern era, the web and mobile application users demand instant access to the data and information whenever any change or update occurs in the cloud database while showing the data automatically on their screens without refreshing their client application. To achieve these requirements, Fig 2 shows the architecture for data-driven applications, based on GraphQL, in a commonly used BaaS configuration [23].

The architecture centers on the broadcasting of real-time data from the serverless backend to all the clients or broadcasting data among multiple clients. The main concepts include Client, Operation, Action, Schema, Proxy, Resolver and Data Source.

- A **Client** is designed to quickly build a UI that fetches the data from the cloud and can be used with any JavaScript front-end. GraphQL operations are defined in a client. The client performs an appropriate authorization wrapping of the request statements before submitting to the proxy for synchronizing. Responses are persisted in an offline store and the write requests are made in a write-through pattern. The client submits the operation request to the proxy along with an identity context and credentials. Identity represents the caller, based on a set of credentials, which must be sent along with every request to the proxy.
- **Operation** includes the following: 1) A read only fetch call to the data. 2) A write of the data followed by the fetch. 3) Long lived connection that receive the data in response to events.
- **Action** is a notification to the connected subscribers which is the result of a write operation. The client becomes a subscriber through a handshake process.
- **Schema** defines the capabilities of an API to which the client application communicates. All requests are validated with the schema.
- **Proxy** is a component that processes the received requests and maps them to logical functions for data operations or triggers. It also manages the conflict detection and resolution strategies.
- **Resolver** is a function that converts the payload to the underlying storage system protocol and executes on a caller authorization.
- **Data Source** is a persistent storage system, to which the API interacts with. The application state is managed by the system, defined in a data source.

### State-of-the-art approaches

While the information technology (IT) industry is moving into a wide array of cloud platforms, numerous researches related to the cloud application development are available in the literature.

#### Model-driven frameworks for cloud computing

Fylaktopoulos et al. [25] propose a model-driven framework for the rapid development of advanced cloud applications. However, it has some shortcomings in terms of development methodology such as change in the programming language by developers, the set of tools used in the development and the security issues. In order to address these issues, Almorsy et al. [26] propose a model-driven approach for securing multitenant applications. Their proposed approach captures and verifies the security issues of multiple tenants as well as the service providers at runtime, without customizing the underlying service or application. Similarly, Mendoza et al. [27] propose a cloud platform using a model-driven approach that develops, executes and hosts rich mobile applications and based on the third-party backend services such as Yahoo weather, Instagram, Twitter REST etc.

#### Data-driven applications

Various researches, related to data-driven applications, have been found in the literature. For instance, Fujimoto et al. [28] propose a dynamic data-driven system to track a vehicle. The approach in [28] tracks a vehicle’s movements from the live video and image data to predict the future locations and data dissemination over a wireless network. Furthermore, it selects a suitable prediction model dynamically based upon the currently available data. Similarly, Suh et al. [29] propose a dynamic data-driven transportation system that estimates and predicts the performance measures through simulation. In addition to the dynamic data-driven approaches of [28] and [29], Liu et al. [30] present a data-driven composition approach for the service oriented situational web applications. They propose a technique that extracts the information from various sources to abstract the service capabilities with set tags which are then exploited by the planning techniques to achieve the desired goals.

In the context of data-driven approaches for the development of different mobile applications, the work proposed by Deka [31] provides two categories. In the first group, the data is captured seamlessly in background and a human interaction is used for exploring the target application. On the other hand, the second group uses some automated agent leveraging human interaction mechanism. In the context of data synchronization, Liu and Lai [32] propose an approach for the mobile peer to peer systems which exploits the dynamic inverted data indexing with the group-based data-driven consistency management. Similarly, the approach of Kumar et al. [33] is based on a dashboard framework which amalgamates the data from various sources (i.e. Flurry, JSON, Google Analytics and Excel files) to create a customizable user interface.

As far as the data-driven applications for cloud environments are concerned, Zhu and Liu [34] propose a data-driven automatic configuration tuning tool for cloud systems. Their proposed tool auto-tunes the performance by utilizing a machine learning classification model. The experimental results have shown that the tool in [34] can efficiently tune the system performance, particularly for the high dimensional inputs. Similarly, Gianniti et al. [35] propose a model-driven framework (D-SPACE4Cloud) to enhance the quality of big data applications in cloud. The objective is to fill the gaps in model-driven engineering with respect to data-intensive applications development in cloud environments, for minimizing the deployment cost and satisfying QoS (Quality of Service) constraints. The proposed tool in [35] provides capacity scheduling of big data clusters in the cloud that efficiently supports data-intensive applications.

In addition to the works in [34] and [35], Perez-Palacin et al. [36] propose a UML Profile for the design, quality assessment and deployment of data-intensive applications. Their proposed domain specific UML Profile considers three abstraction levels (DPIM, DTSM and DDSM) for the development of data-intensive applications. The DPIM (Data-intensive application Platform Independent Model) defines the main architecture of data-intensive applications. The DTSM (Data-intensive application Technology Specific Model) includes a specific technology, e.g., Apache Hadoop for the quality assessment of data-intensive applications. Finally, the DDSM (Data-intensive application Deployment Specific Model) enables the automatic deployment of data-intensive applications to public or private clouds. Similarly, Tolosana-Calasanz et al. [37] present a PetriNet based model-driven approach for continuous data flow applications, executed in cloud. Their proposed methodology specifies functional and non-functional requirements, along with the specification of involved computational resources, to provide corresponding views such as data flow, control flow and resources.

#### Serverless computing

With the paradigm shift to serverless computing (also known as serverless cloud computing), several research studies have targeted the serverless computing. The following three paragraphs have discussed some of those.

Dean et al. [38] present a generic “unicast to multicast proxy toolkit” to implement an XMPP (Extensible Messaging and Presence Protocol) proxy which dynamically supports the creation and operation of multi-user conversations in a serverless environment. They provide the evidence of their approach by showing the success of XMPP protocol in a wireless mobile environment. Similarly, Perez et al. [39] propose a framework to run Docker containers in AWS lambda serverless platform. Their proposed approach opens new methods to adopt serverless computing in the scientific use cases that were formerly not easily exploitable. They also provide the facility to explore new programming languages in a serverless platform.

Yan et al. [40] present a chatbot prototype using serverless platform, which is based on some function sequences, to coordinate with the cognitive services in the Watson Developer cloud so that the chatbot can interact with the outside services. Sampe et al. [41] propose a data-driven middleware for object storage using serverless computing. The proposed functions in [41] capture and operate over objects, as they are read or write to an object store. Furthermore, they use OpenStack Swift to implement a prototype of their serverless framework which stores a huge volume of data through RESTful API. Similarly, Malawski et al. [42] present the key features of serverless infrastructures which are designed primarily for processing background tasks of Web, IoT and stream processing applications. They also discuss the possible ways of executing scientific workflows and develop a prototype workflow execution functions using AWS lambda, Google cloud functions and HyperFlow workflow engine. Nastic et al. [43] propose a platform that supports the real-time data analytics in edge computing applications. They introduce a serverless execution model which allows a stable development of the real-time analytics functions, thus freeing developers from the underlying infrastructure at the edge.

Gribaudo et al. [44] propose a performance evaluation modeling framework, for lambda architecture-based applications, to support the design and decision process. The authors in [44] also provide a domain specific language for hiding the complexity of an evaluated model. The work of Ast et al. [45] propose an approach for the enabling of self-contained web components in serverless computing. Generally, the functionality of web components consists of presentation and business logic. Their proposed approach deploys the business logic of web component as cloud hosted functions which can easily be integrated into existing websites, thereby reducing the cost and time. However, their proposed solution cannot be merged into current cloud providers due to some user privileges for security and privacy requirements.

Table 1 compares various state-of-the-art approaches with the proposed method in terms of various attributes. These attributes are Serverless Computing, Data-driven Applications, Frontend Prototype, Backend, Validation on Cloud Providers, Modeling Approach and Tool Support, as shown in the first row of Table 1.

The following points can be deduced from Table 1:

- No state-of-the-art study provides a tool support for data-driven applications in serverless cloud computing in the form of a transformation engine. However, different researches provide various solutions using some data-driven based frameworks (second row of Table 1). For example, the frontend prototype provided in [33] focuses on the creation of a single dashboard user interface (pie chart, column chart, line chart) for the data analytics without any modeling and tool support. Similarly, the work in [31] provides the frontend prototype for mobile applications. Moreover, the research study [9] provides a prototype tool without the modeling support for data-driven web applications using MySQL. However, the proposed prototype tool in [9] does not support the real-time data updates in a distributed environment.
- A few research studies such as [28] and [29], targeting the data-driven applications, focus on a specific application domain without providing any modeling and tool support (third row of Table 1). On the other hand, there are some works [34–37] for data-intensive applications which also provide backend solutions with modeling and tool support, focusing only on performance, design, quality assessment and deployment in cloud systems. However, these studies [34–37] lack a serverless architecture for application development. In order to target the serverless computing, the work in [41] provides a solution for the data-driven serverless stateless functions through REST API without any modeling and transformation tool (seventh row of Table 1). Similarly, the work in [38, 39, 42] and [43] support a solution for the backend without providing any modeling and tool support (sixth row of Table 1). However, the work in [44] also provides the modeling and tool support but only for evaluating the performance of lambda-based applications (eighth row of Table 1). Finally, the second last row of Table 1 shows that the work in [45] provides the frontend prototype for deploying the business logic of web components without any modeling and tool support.
- To summarize, there is a lack for the solution/framework that can simplify the behavioral aspects (the low-level implementation) of data-driven applications based on GraphQL in serverless cloud computing including both frontend and backend. Consequently, this study proposes a new UML profile, termed as UMLPDA, to support the modeling of both frontend as well as backend requirements for the data-driven applications with the real-time data update in a serverless cloud computing environment.

## UMLPDA: Unified modeling language profile for data-driven applications

**MDA (Model-driven architecture)** is a renowned software design methodology to abstract the complexity of software development [46]. In an MDA paradigm, UML models can be customized and extended using a UML profile diagram for some specific application domains and are based on various elements [12]. These elements are Stereotypes (allow the designer to increase UML vocabulary), Tag values (used to extend the UML properties so that one can add additional information in the specification of a model element) and Constraints (to specify those conditions that must be true at all the time). All of these (stereotypes, tag values and constraints) are applied to some specific model elements such as Classes, Operations, Attributes, and Activities.

Consequently, the proposed UMLPDA profile provides some appropriate stereotypes to model the frontend as well as backend requirements of data-driven applications through GraphQL architecture in a serverless cloud computing environment. It consists of user interface concepts, backend GraphQL API schema concepts and behavioral concepts of the application. The proposed UMLPDA is developed in UML tool Papyrus, based on Eclipse [47].

### User interface concepts

**Fig 3:**
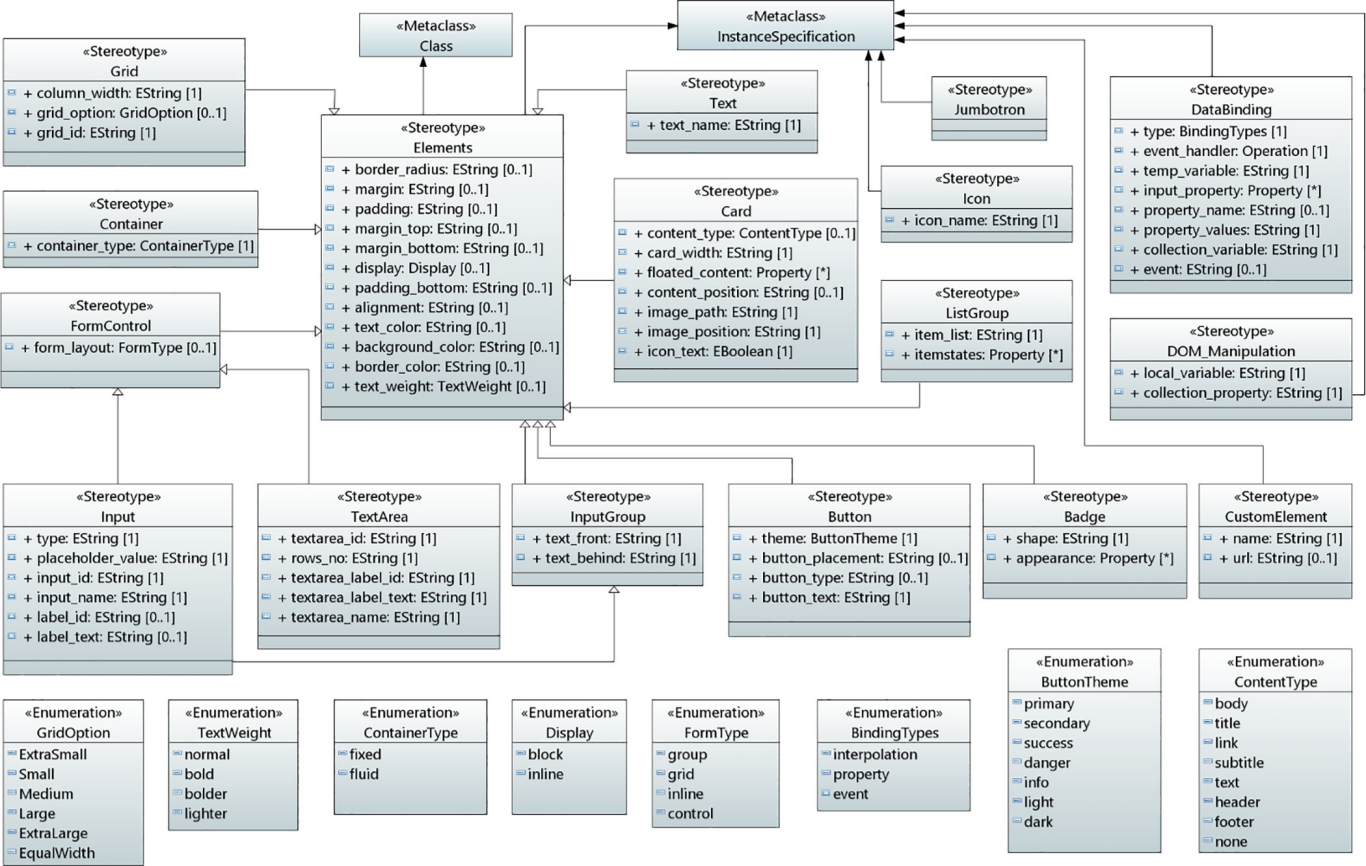
User interface concepts in UMLPDA.

To develop a fully functional data-driven applications in serverless cloud computing, user interface is one of the key requirements, which provides an interaction between the user and a cloud system/service through a network. Due to the complexity of business logic, the user interface concepts, and the application functionality are designed separately. The user interface concepts include various components to send the data such as input, input group, text area and form control. List, card and badge components are used for displaying the data while data binding is essential to bind the data retrieved from the cloud data source. Fig 3 shows the modeling of user interface concepts. It consists of seventeen stereotypes, eight enumeration types and two base classes. A brief description of these stereotypes is given in the following:

**Elements** stereotype defines the common properties, pertaining to the elements of user interface. It is extended from the standard UML Class and Instance specifications meta-classes. It has the following tag values: The “border_radius” tag value is used to define the radius of the elements’ corner. Similarly, the “margin” and “padding” tag values set the spacing of elements. Moreover, tag values “margin-top” and “margin-bottom” specify the margin for top and bottom sides of an element respectively. In order to define the display property of an element, the tag value “display” is used. Similarly, other tag values such as “alignment” sets the horizontal and vertical alignment of the element. The “text_color”, “background_color” and “text_weight” specify the color, background color and font weight respectively. With the help of aforementioned tag values, “Elements” stereotype facilitates the development of a correct UI through the proposed UMLPDA.

**Container** stereotype is used to center and horizontally pad the contents of the site. It consists of one tag value “container_type”, which defines the width of the viewport. This stereotype manages the correct layout of an element.

**Grid** stereotype defines the layout and aligns the contents by adding a series of rows and columns. It comprises of three tag values: “column_width” (defines the width of column in percentages), “grid_option” (specifies the screen size in pixels) and “grid_id” (represents unique id of grid element).

**Card** stereotype is used to display the contents and contains seven tag values. The tag value “Cardtype” defines the content type supported in the card including text, images, links etc. The tag value “card_width” defines the width of the card. Similarly, tag value “floated_content” defines the positioning and formatting content. The tag value “Color_text” specifies the text color on the card. The “Image_path” defines the path of the image to be displayed on the card. The tag value “image_position” illustrates the position of the image. The tag value “icon_text” is set to true if the data on the card contains an icon image.

**InputGroup** stereotype adds text, button or button groups and consists of two tag values: “text_front” and “text_behind”. The tag value “text_front” is used to add the help text in the front of input while “text_behind” specifies the text behind the input.

**Input** stereotype specifies a form control for input. For Input stereotype, the tag value “type” defines the input type. Similarly, the tag value “placeholder_value” specifies the value to be written as a placeholder in input. The tag values “input_id” and “input name” describes the id and name of the input element respectively.

**TextArea** stereotype includes textarea_id, rows_no, textarea_label_id, textarea_label_text and textarea_name. The TextArea stereotype along with the Input stereotype facilitate the designer to accurately form control elements.

**Button** stereotype defines the button element and comprises of several tag values that are used to define the style of a button. For example, the tag value “theme” specifies the button theme while the tag value “button_text” defines the text to be placed on the button. Similarly, the “button_type” sets the type of the button and the “button_placement” defines the button position.

**Badge** stereotype defines a link or button to provide a counter and adds a badge element to the user interface. It contains two tag values: shape and appearance. The tag value “shape” defines the shape of the badge (e.g rounded etc) while the “appearance” specifies the appearance.

**ListGroup** stereotype defines a series of contents and facilitates the development of a list element. The tag value “Items_list” defines the items to be added in the list. The tag value “ItemStates” defines the item states such as active and disabled.

**CustomElement** stereotype defines a new DOM element. The tag value “name” specifies the custom element name and the tag value “url” defines the path to be navigated.

**FormControl** stereotype specifies the layout and style of the form controls. The tag value “form_layout” vary the layout of the form control.

**Jumbotron** stereotype extends the entire viewport to show the messages on the site.

**Text** Stereotype specifies the text to be placed on headings, icons etc. The tag value “text_name” presents the text to be appeared on the screen.

**Icon** Stereotype places the icons inside the text and makes it easier to add an icon at the desired location on user interface. The tag value “icon_name” specifies the name of the icon.

**DataBinding** stereotype controls the data flow in the application between a component and its view template. It comprises of several tag values. The tag value “binding type” selects the type of binding from enumeration class BindingTypes which presents three types of binding: event, interpolation and property. The “event_handler” binding defines an operation to be performed on a specific event. Event is the occurrence of a certain action. The tag values “temp_variable”, “input_property”, “property_name”, “property_values” and “collection_variable” specify the values to be used for displaying data received by the client. This stereotype facilitates the development of exact data required by the user interface elements.

**DOM_Manipulation** stereotype is used to bind the application data to the attributes of HTML DOM elements. It has two tag values (“local_variable”, “collection_property”) which are used to change either the layout of application or the behavior of DOM element.

Each of the aforementioned stereotypes is extended to UML Class and Instance Specification meta-classes. Furthermore, eight enumeration classes are also defined such as TextWeight, GridOption, ContainerType, FormType, ContentType, ButtonTheme, BindingTypes and Display. Each of these enumeration classes contain literal values. For example, TextWeight defines the font weight which can be one of these four literal values: “normal”, “bold”, “bolder”, “lighter”. GridOption enumeration class defines the corresponding screen sizes and can be one of these six literal values: “ExtraSmall”, “Small”, “Medium”, “Large”, “ExtraLarge” and “EqualWidth”. Similarly, all other enumeration classes also define various values as per their enumeration type. Furthermore, the application of all these stereotypes is given in “Validation” section of this article.

### GraphQL API schema concepts

**Fig 4:**
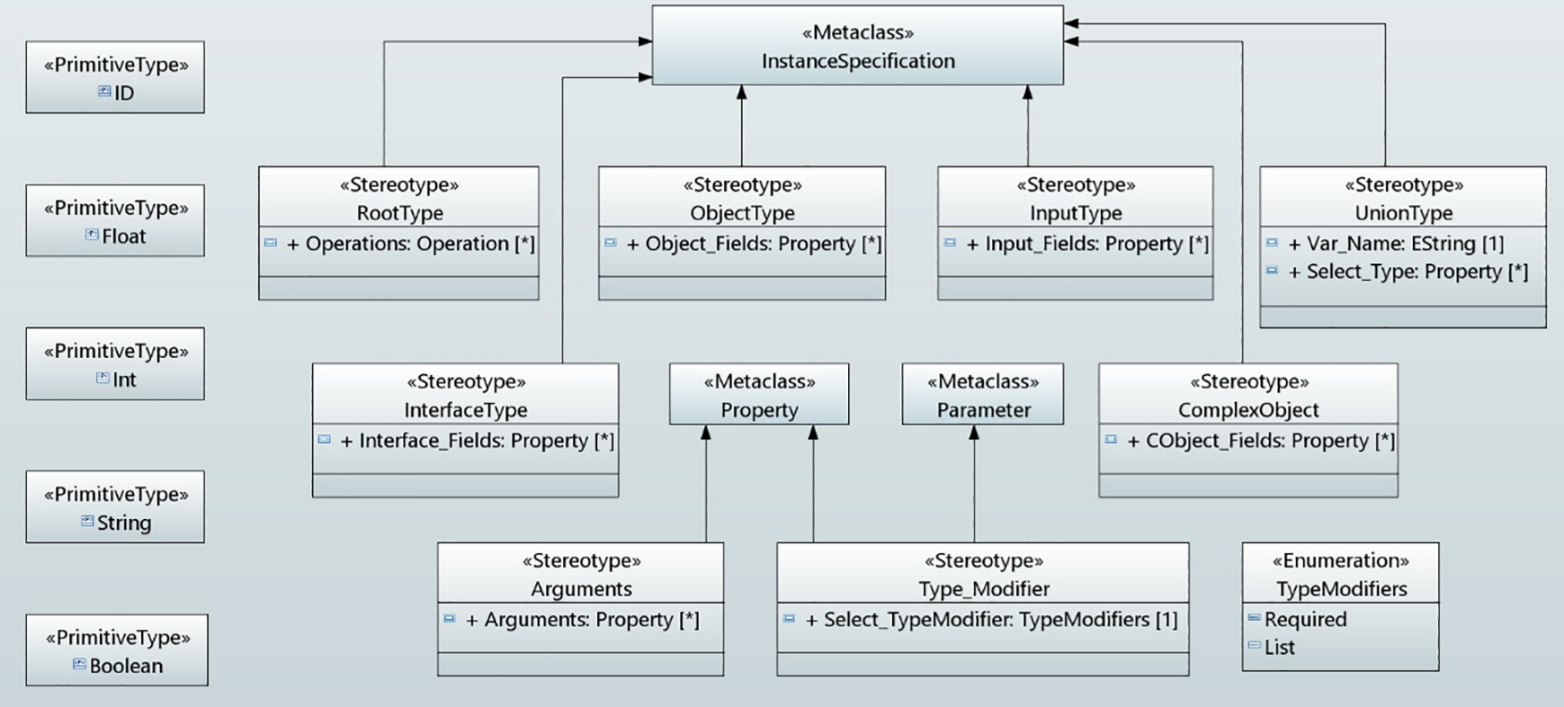
GraphQL API schema concepts in UMLPDA.

In the context of data-driven applications, a GraphQL API schema defines the GraphQL server’s API, so that the client knows which operations can be carried out by the server. The schema defines the object types (user defined types) and fields for representing the data which is retrieved from the API and the root types (query, mutation and subscription) to define a certain set of operations permitted by the API. Query type defines the read operations, mutation type illustrates the write operations and subscription enables a server to send the data to its clients when a mutation occurs. These operations can run a request through a GraphQL server. It also encompasses several other types such as input type, union type and others. Fig 4 shows the concepts, defined for GraphQL API schema modeling, used in the serverless backend of data-driven applications.

**RootType** stereotype defines the entry point of an application. It has one tag value “Operations” which is used for the data fetching. This stereotype smoothly develops the main operations for data fetching for root types.

**ObjectType** stereotype defines an object that can be fetched from the defined service and facilitates the development of an object type in the schema. The tag value “Object_Fields” defines the fields to represent data on that object through a property type.

**InputType** stereotype is similar to the object type. The tag value “Input_Fields” defines the fields for representing data on an input object through a property type. This stereotype defines the input type in the schema for complex objects.

**InterfaceType** stereotype defines an abstract type that includes a certain set of data fields which a type must include to implement the interface. The fields in the interface type are defined with the tag value “Interface_Fields”. This stereotype supports an object or set of objects that is to be returned and those objects are of different types.

**UnionType** stereotype is very similar to InterfaceType except that they do not specify any fields for data representation between the types. It comprises of “Var_Name” which specifies the name for the union type while the “Select Type” defines the concrete object types.

**ComplexObject** stereotype defines the complex data such as videos or images as part of their structure. The tag value “CObject_Fields” encompasses the fields, required to define the complex object type. The above six stereotypes (RootType, ObjectType, InputType, InterfaceType, UnionType and ComplexObject) are extended from Instance Specification meta-class.

**TypeModifier** Stereotype defines the special group of types. It is extended from UML Property and Parameter meta-class. It has one tag value “Select_TypeModifier” of type enumeration. This attribute selects the desired modifier type from the enumeration. An enumeration class TypeModifier is defined having two literal values: Required and List. The literal value “Required” presents a type as non-null by adding an exclamation mark after the type name while the “List” indicates that the field will return an array of that type.

**Arguments** stereotype defines the arguments within the data fields or operations. It is extended from Property meta-class. The tag value “Arguments” take the arguments used in the fields. Five primitive types have been defined which represent the scalar type. These are ID, Int, Float, String and Boolean.

### Behavioral concepts of data-driven applications

**Fig 5:**
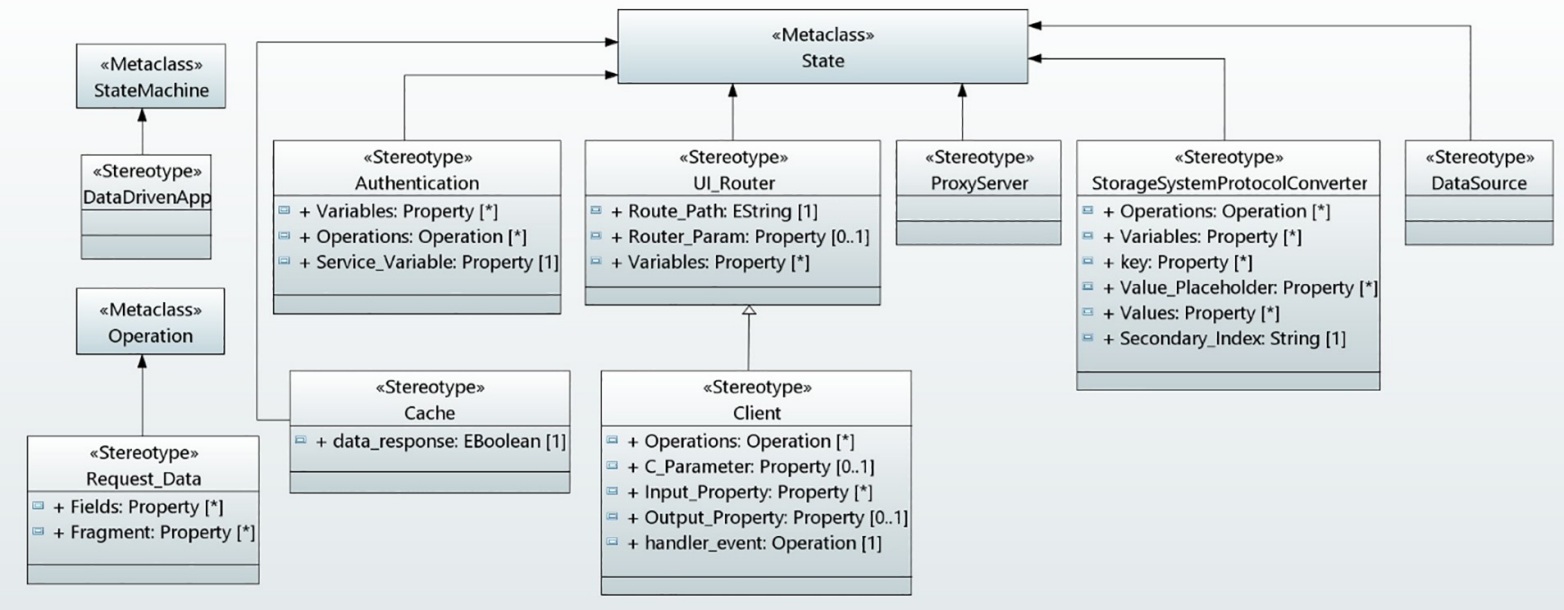
Behavioral concepts of data-driven applications in UMLPDA.

This section describes the behavioral concepts of data-driven application in serverless cloud computing in a state machine diagram which is based on GraphQL architecture, as shown in Fig 5.

For the data communication between a client application and GraphQL server on a cloud and retrieving the real-time updates in an application requires to develop the underlying functionality of the data-driven applications. The behavioral concepts include user authentication, a client application, a GraphQL server, resolver functions for writing and fetching the data from the cloud data sources, a cache for storing and updating the data, and data operations in the client application which includes read/write and subscribe operations to communicate with the GraphQL API. The client application receives real-time data updates through the subscribe operations whenever new data arrives at the backend cloud data source. The underlying infrastructure concepts are managed by the cloud provider itself. The modeling concept comprises of several stereotypes, required to perform an application behavior. The details are as follows:

**DataDrivenApp** stereotype represents the data-driven application. Therefore, it is extended from StateMachine meta-class.

**Authentication** stereotype is responsible for the user authentication to secure and protect the data. To manage the identities tag value, “Variables” and “Operations” are used to define various variables and operations used. The tag value “Service_Variable” specifies the instance of the service to be used for authentication.

**UI_Router** stereotype manages the transitions between states of an application. The tag value “Route_Path” defines the navigation path to the router. Similarly, the “Router_Param” specifies the parameter to be passed during router navigation. Tag value “Variables” specifies the variables used in the navigated state.

**Client** stereotype is defined to communicate with the backend cloud data sources and ultimately binds the data to the frontend user interface element. The tag values “Operations” specifies the operations used for data communication with the backend GraphQL API. These operations basically create read, write and subscribe operations in a client. The data is supplied to the component through an injectable service class. The tag value “C_Parameter” defines a parameter that injects a dependency in a component. The “Input_Property” binds the property within a child component to receive a value from the parent component. The “Output_Property” binds a property of a component to send data from the child component to the parent component. The tag value “handler_event” defines the event handler which is of type Operation.

**Cache** stereotype stores the data and updates the corresponding cache on the arrival of a new data from the cloud data source. The tag value “data_response” is defined which becomes true if the data is found in the cache.

**ProxyServer** stereotype is a GraphQL engine on a cloud that processes some client requests and map them to some logical functions for the corresponding data operations.

**StorageSystemProtocolConverter** stereotype defines the resolver functions on a cloud which converts the payload data to an underlying storage system protocol and executes on a caller authorization. These resolver functions connect the fields in the GraphQL schema to a cloud data source. It includes the following tag values: Operation, Variables, Key, Value_Placeholder, Values and Secondary_Index. The tag value “Operation” specifies the NoSQL database operation, required to be performed on a cloud data source. Similarly, the tag value “Variables” defines the remaining attributes of the item to be placed into a database. The “Key” denotes the key of the item in a database. The “Value_Placeholder” defines the query expression. “Values” defines the substitution for expression attribute value. Finally, the “Secondary_Index” specifies the index to a query that can be scanned in addition to the primary key index. With the help of these tag values, this stereotype facilitates the development of a request and responses to mapping templates.

**DataSource** stereotype stores the data in a cloud database. The above seven stereotypes are extended from State meta-class.

**Request_Data** stereotype is defined to create the requested data in the form of data structure for interacting with the GraphQL API to read/write/subscribe the data. It is extended from Operation meta-class and encompasses two tag values: “Fields” define the data which must be fetched from the cloud data source while “Fragment” can construct a set of fields. Consequently, this stereotype provides a convenient way to develop some appropriate data request operations.

The practical applicability of all the aforementioned stereotypes, described in this section, with three case studies will be illustrated in “Validation” section of this article.

## Transformation engine

This section provides implementation details of the transformation engine. Firstly, the transformation rules are developed to generate Angular2 and GraphQL code from UMLPDA. Then, the architecture of UMLPDA transformation engine is described.

### Transformation rules

In MDA, the transformation of higher-level source models to the target platform specific models or low-level code is generally carried out through transformation rules [48] either employing M2T (Model to Text) or M2M (Model to Model) transformations. The generated code from M2T transformation reduces the development time and effort as well as minimizes the risk of introducing errors during the development process. This section discusses the transformation rules which are used to map UMLPDA into Angular2 and GraphQL. These rules produce a target frontend and backend code for data-driven applications through a transformation engine.

#### Transformation rules for user interface

Table 2 highlights the transformation rules for transforming UMLPDA concepts into the corresponding Angular Html Template concepts.

**Table 2:** Transformation rules for user interface.

Sr. No	UMLPDA Concepts	Corresponding Html Concepts	Description
1.	**Container**	Viewport	Container→viewport
2.	**Grid**	Grid	Grid→grid
			grid_option→grid breakpoints
			column_width→width
3.	**Elements**	Component	Elements→component
			margin→element spacing
			border_radius→ elements corner
			margin_top→top
			margin_bottom→bottom
			padding→spacing
			alignment→position
			display→layout
			text_weight→fontweight
4.	**Input-Group**	input-group	Input-Group→input-group
	**Input**	Input	Input→input
			text_front→input-group-prepend
			text_behind→input-group-append
			form_type→form-control
			placeholder_value→required
			placeholder
			input_id→id
			input_name→name
5.	**Button**	Button	Button→button
			theme→button styles
			button_text→text
6.	**List-Group**	list-group	List-Group→list-group
			Items_List→list-group-item
			ItemStates→list-group-item→Active
			Items
7.	**Card**	Card	Card→card
			Card_width→width
			content_Type→ContentType
			floated_content→floatPosition
			content_position→position
			color_text→color
8.	**Badge**	Badge	Badge→badge
			shape→badgeshape
9.	**Icon**	Icon	Icon→icon
			Icon_name→name
10.	**TextArea**	Textarea	TextArea→textarea
			textarea_rows→rows
			textarea_label_text→label
11.	**DataBinding**	Binding types	Types→BindingTypes
			event_handler→Operation
12.	**DOM_Manipulation**	Directives	DOM_Manipulation→StructuralDirectives

#### Transformation rules for backend GraphQL API schema

The transformation rules for transforming the UMLPDA concepts into the corresponding GraphQL concepts are summarized in Table 3. Only RootType and ObjectType rules are discussed in this section along with the type modifiers and arguments.

**Table 3:** Transformation rules for backend GraphQL API schema.

Sr. No	UMLPDA Concepts	Corresponding GraphQL Concepts	Description
1.	**RootType**	Query, Mutation and Subscription type with operations	Operations→Request Operations
2.	**TypeModifier**	Required or null (Parameters or Fields)	TypeModifier→Fields
			TypeModifier→Parameters
3.	**ObjectType**	User-defined types with data fields	Object_Fields→Fields
4.	**Arguments**	Contains the arguments in the data fields	Arguments→Fields

#### Transformation rules for data-driven applications behavior

The transformation rules for transforming UMLPDA concepts into the corresponding Angular 2, GraphQL and VTL (Velocity Template Language) code are shown in Table 4.

**Table 4:** Transformation rules for data-driven application behavior.

Sr. No	UMLPDA Concepts	Corresponding Angular2, GraphQL and VTL (Velocity Template Language) Concepts	Description
1.	State UI_Router	Contains the routing state and path for the stereotype UI_Router (Angular2 Concept)	UI_Router→Component
2.	State Authentication	Contains the user authentication for the stereotype Authentication (Angular2 Concept)	Authentication→CognitoUserPool for token-based authentication.
3.	State Client	Contains the state operations for the stereotype Client (Angular2 Concept)	Client → StateOperations (Client mapped to the state operations)
4.	State StorageSystemProtocolConverter	Contains the resolver functions for the stereotype StorageSystem ProtocolConverter (VTL Concept for cloud data)	StorageSystemProtocolConverter→ Resolver function with data conversion for requested data
5.	State DataSource	Contains the operation to be performed for stereotype DataSource on cloud	DataSource → Database Table
6.	Request_Data Operation	Contains the operations which defines the data structure for the Request_Data (GraphQL Concept)	Request_Data→Operation

We have developed several UMLPDA concepts for logically representing the concepts of data-driven applications. In order to develop some appropriate transformation rules, we perform mapping between the UMLPDA concepts and the corresponding HTML/Angular2, GraphQL and VTL concepts as given in Tables 2, 3 and 4 respectively. Particularly, for the generation of user interface, the mapping between UMLPDA modeling elements and the corresponding HTML/Angular2 constructs is straightforward as given in Table 2. For example, the UMLPDA stereotypes like container and grid are directly transformed to the corresponding HTML constructs like viewport and grid. Similarly, for GraphQL API schema (Table 3), the stereotypes like RootType and ObjectType are transformed to query/mutation types with operations and user-defined datatypes respectively.

On the other hand, few direct transformation rules from the UMLPDA stereotypes to behavioral logic are given in Table 4. However, in addition to such direct rules, several indirect rules are also developed and implemented in a transformation engine for the generation of data-driven application behavior. For example, we implement the rules regarding GraphQL read/write/subscribe operations in transformation engine for Request_Data stereotype in order to achieve a seamless interaction of GraphQL client with GraphQL API. Similarly, we implement the rules for resolver functions in transformation engine for StorageSystemProtocolConverter stereotype in order to connect the GraphQL API schema type fields to a data source and returning the data response. Such indirect rules cannot be systematically included in tabular form, therefore, we do not include these indirect and low level rules here. In fact, these details are available in source code [49].

### Transformation engine architecture

**Fig 6:**
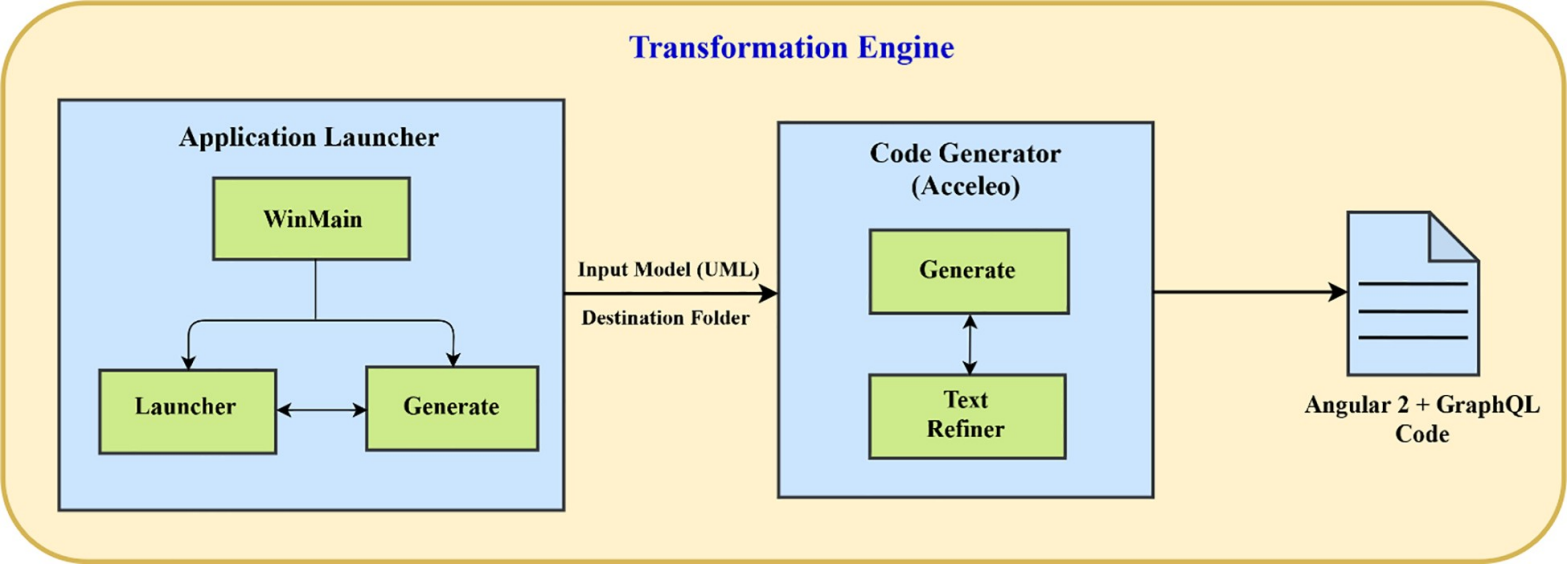
Transformation engine architecture.

After defining the transformation rules, we developed a transformation engine by implementing transformation rules to generate Angular 2 and GraphQL code from high level models. The transformation engine architecture is shown in Fig 6.

The tool used for model to text transformation is Acceleo. The transformation engine comprises of two key components: 1) Application Launcher and 2) Code Generator.

#### Application launcher

The application launcher of the transformation engine consists of three main sub-components which are WinMain, Launcher and Generate. Particularly, WinMain provides the transformation engine user interface implementation. Moreover, various settings required to execute transformation engine are implemented in Launcher sub-component. Additionally, Generate sub-component registers the profile addresses.

#### Code generator

**Fig 7:**
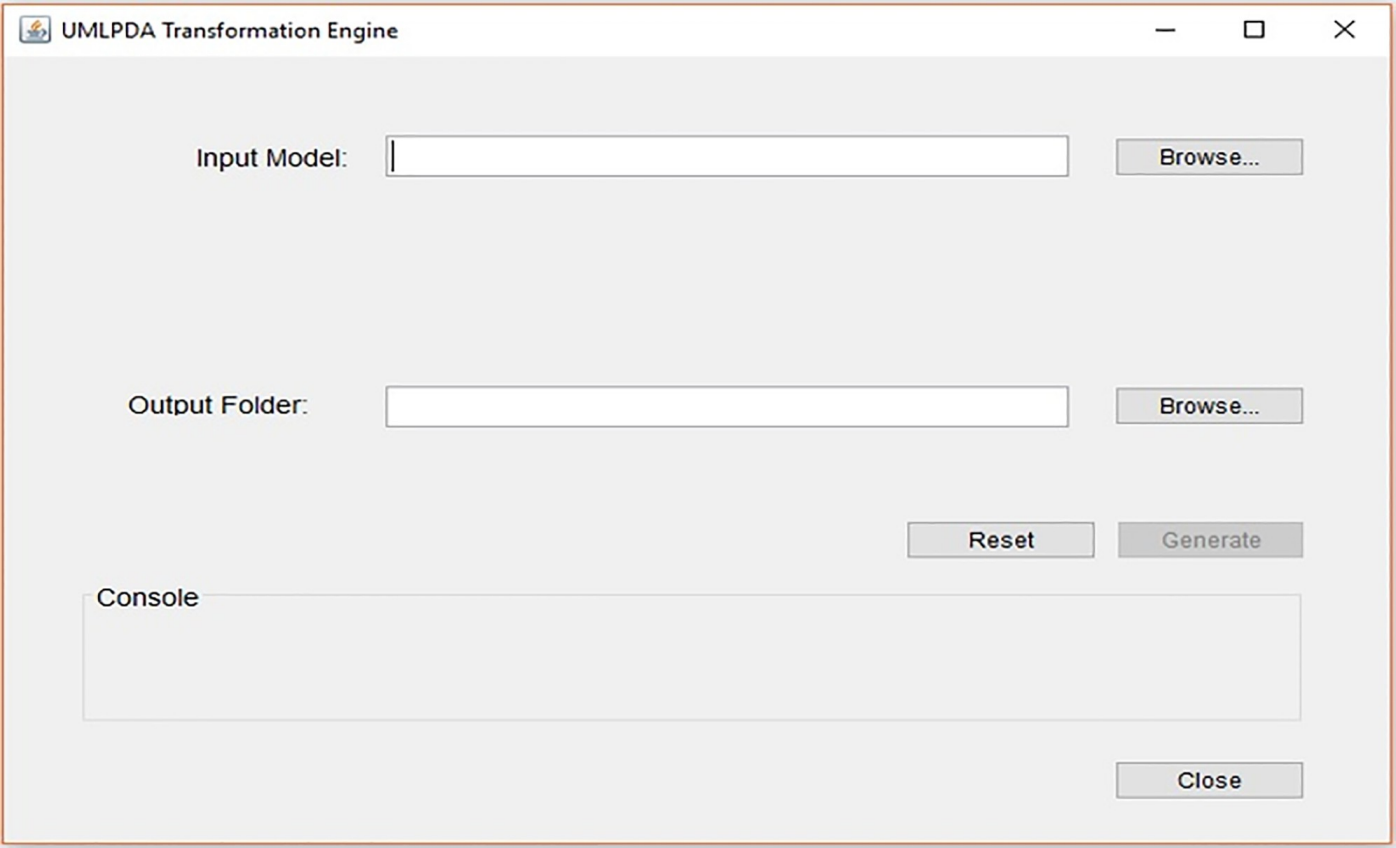
UMLPDA transformation engine input model interface.

This component is responsible for generating Angular 2 and GraphQL code. The input model receives the UML model and output folder receives the destination folder address (where the code files are to be generated) from the application launcher component. The Generate sub-component (Generate.mtl) extracts the desired element from the model. *Text Refiner* is a sub-component that deals with the formatting issues of the generated files. The interface for transformation engine is provide in Fig 7. It takes the UML model and path of the output folder for generating the code from the model using the browse button.

Generate button is provided to generate the required outputs. Console shows the progress of the transformation process. A *Reset* is provided to clear all the fields i.e. input model path, output folder path and console. Finally, a *Close* button is provided to closes the interface from the screen. Here, we have only provided the summary of UMLPDA transformation engine. However, further details like the user manual, sample case studies and the source code can be downloaded from [49].

## Validation

This section presents the applicability of proposed approach with the help of Real-Time Chat Application, Events Calendar Application, and Movies Application. The selected case studies provide the real-time experiences for the application users such as multi-user data synchronization with various device capabilities, manages data with the changing network states and presenting the real-time updates to users. To check the validity of our approach, these case studies are deployed to AWS serverless platform and the results validate the effectiveness of our proposed work. Finally, a discussion is made to compare the proposed approach with the conventional low level implementations.

### Real-time chat application

We first discuss the requirements of Real-Time Chat Application. The corresponding modeling and implementation (transformation and validation) details are then provided.

#### Requirements for real-time chat application

This section contains the requirements for Real-Time Chat Application: (a) all the users must authenticate to use the application and to secure and protect their data, (b) users can see a list of all other users and can start conversation with any of them, (c) only those conversations are visible to which the users are invited or started themselves and (d) users receive a real-time update for new users, new conversations and new messages.

#### Modeling of real-time chat application

**Fig 8:**
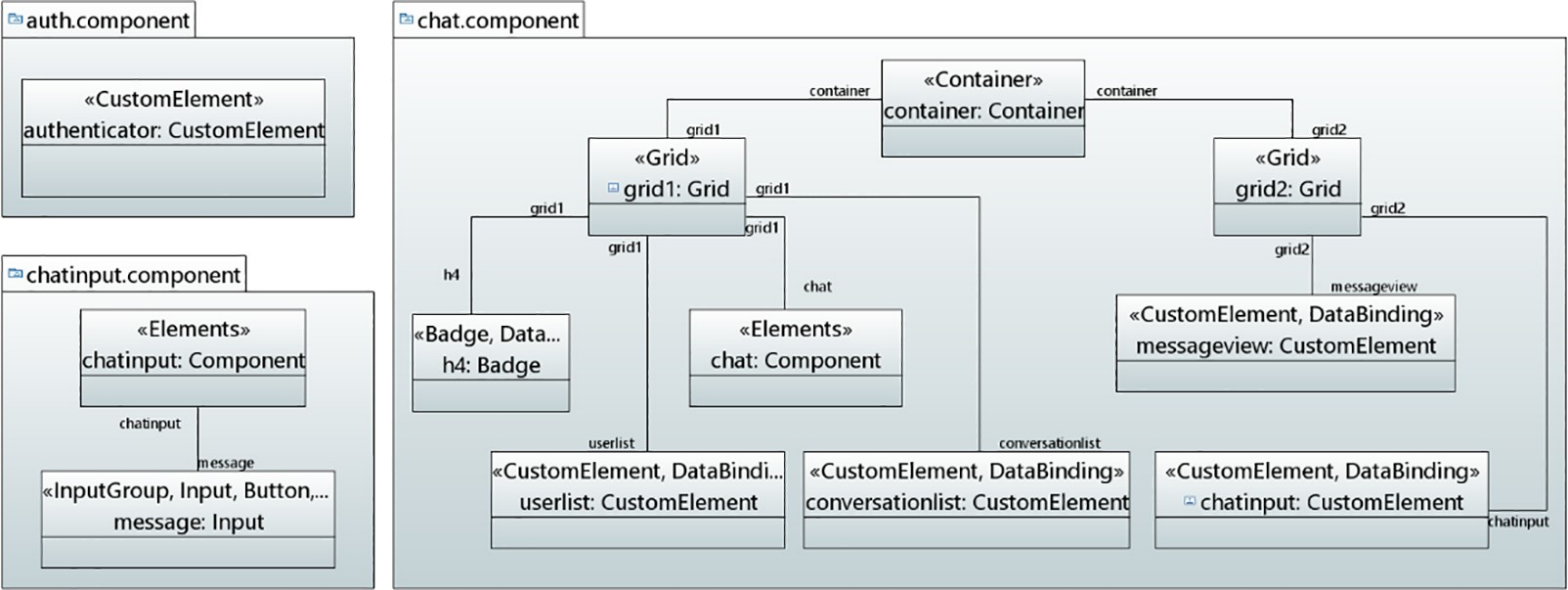
User interface for auth, chat and input components.

**Fig 9:**
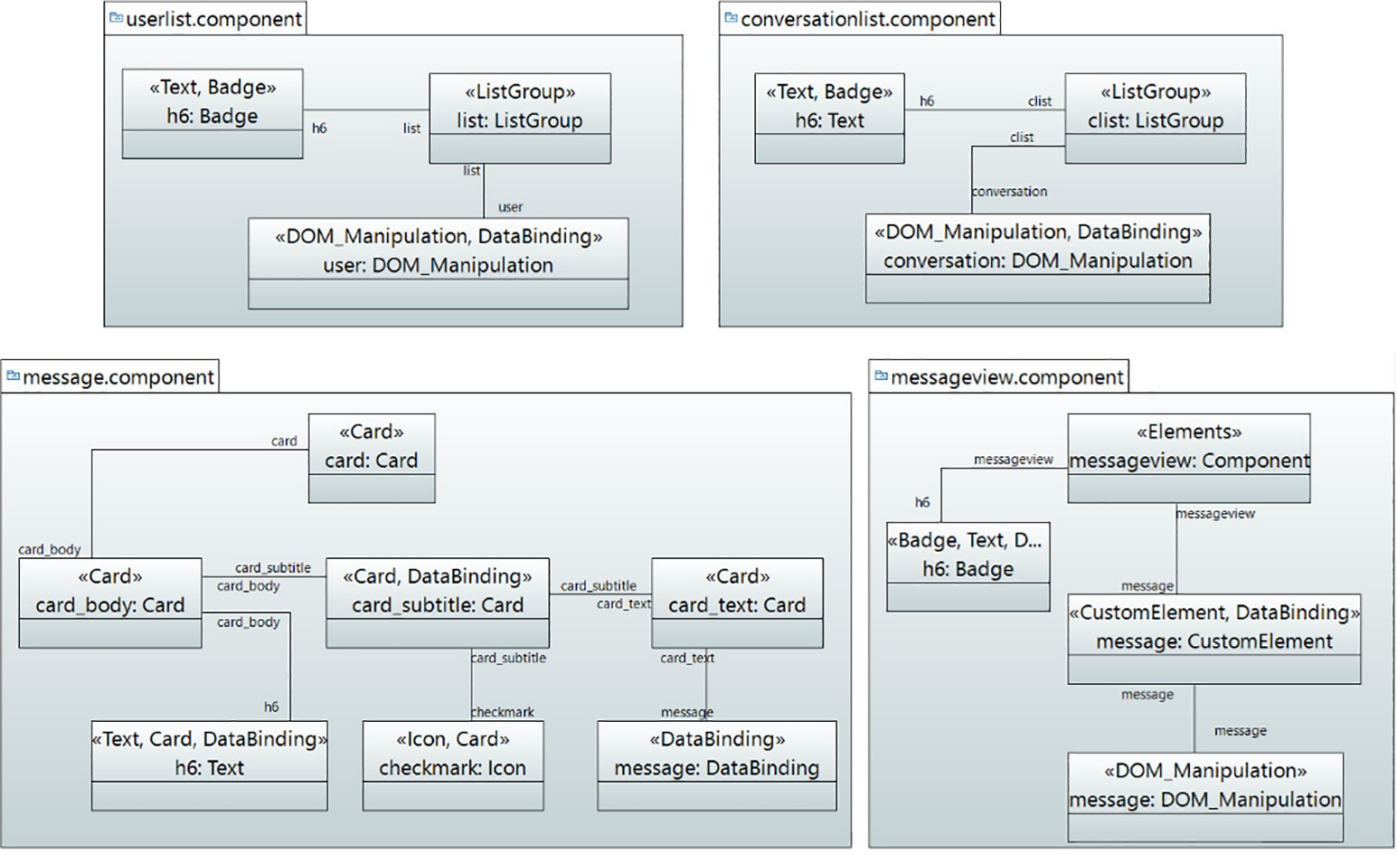
User interface for UserList, ConversationList, message and MessageView components.

*User interface model*. Figs 8 and 9 show the modeling of user interface for Real-Time Chat Application in object diagram. Fig 8 shows the modeling of Auth, Chat and Input Component. In Auth Component, the CustomElement stereotype is mapped to CutomElement which displays the user interface for authentication and handles the sign-up as well as sign-in tasks. The Chat component contains the container, grid, CustomElements and badge elements. Therefore, Container, Grid, CustomElement and Badge stereotypes are applied for adding the elements with layout and styling. Furthermore, it also contains DataBinding stereotype to bind the dynamic data. In the ChatInput Component, the Elements stereotype is applied to add some style to the component. The InputGroup stereotype is applied to add button on the side of input. The Input stereotype is applied to add the input element. Similarly, Fig 9 shows the user interface for UserList, ConversationList, Message and Message View Components.

The UserList component displays users in a list. Therefore, the ListGroup stereotype is mapped to ListGroup that adds users to the user list. The user is retrieved through DOM_Manipulation and DataBinding stereotypes. The ConversationList Component creates conversation. The Elements sereotype is applied to add some styling to the component. The Text stereotype is applied to take the heading text while the ListGroup stereotype is mapped to add the list element. The DOM_Manipulation and DataBinding stereotypes are applied for displaying user conversation. Similarly, the Message Component displays messages through Elements, Grid, CustomElement, DOM_Manipulation and DataBinding stereotypes. Finally, the CustomElement stereotype is applied to add a custom element to the messageview component.

**Fig 10:**
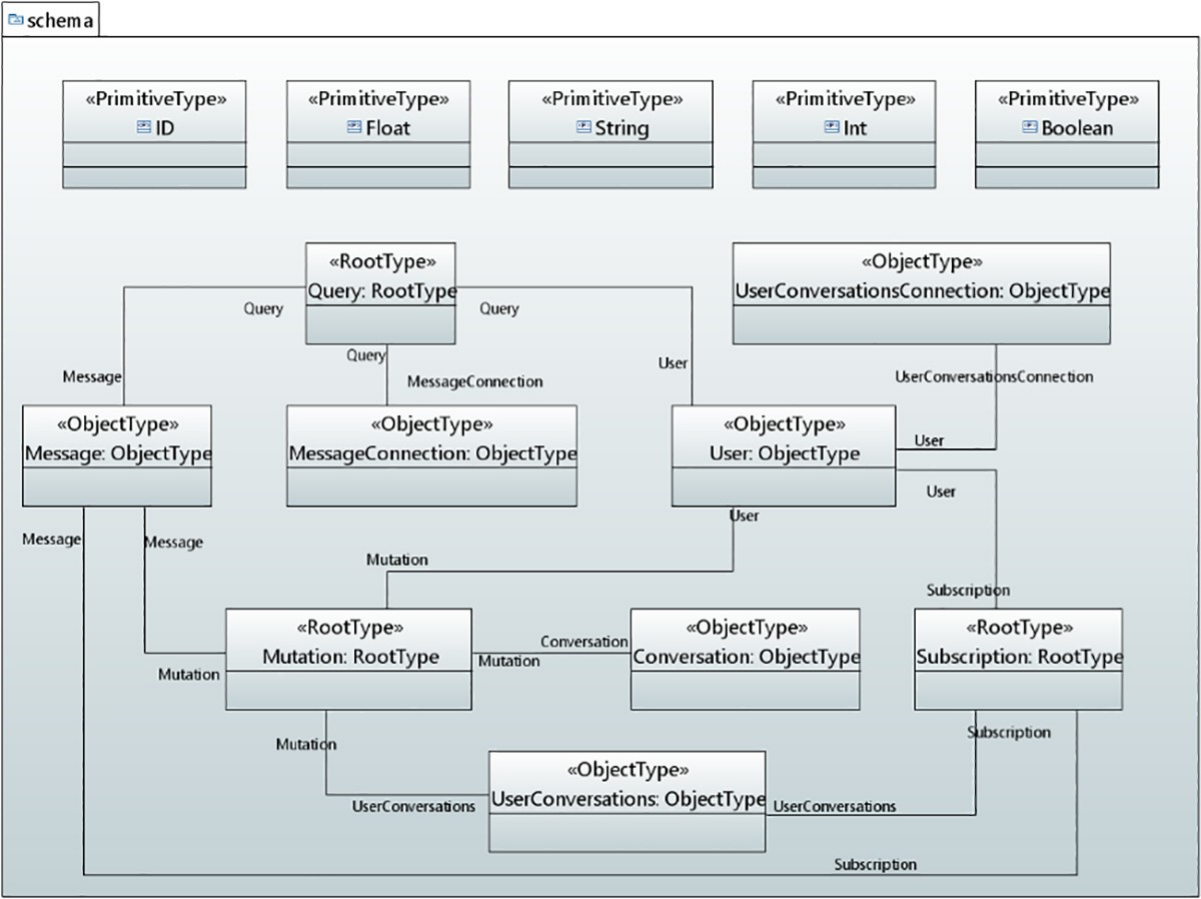
GraphQL API schema for the real-time chat application.

*Backend GraphQL API schema model*. Fig 10 shows the model of the backend GraphQL API schema for Real-Time Chat Application. For this case study, the operations include createUser, createConversation, createMessage, createUserConversations, allUsers, subscribeToNewUser, subscribeToNewMessages etc. Therefore, the RootType stereotype is applied to define the Read, Write and Subscribe operations for root types.

The ObjectType defines the User, Conversation, Message, Message Connection, User Conversations and UserConversationsConnection types. The fields in the ObjectType are defined through ObjectType stereotype such as id, cognitoId and username that represents data for User ObjectType. TypeModifier stereotype is applied to fields in the ObjectType and parameters in the RootType operations depending on whether the data field is required or null. The arguments in the ObjectType fields are defined through Argument stereotype. The Object types and Root type operations are associated with resolvers which are request and response mapping templates for the data read/write from/to a cloud data source.

**Fig 11:**
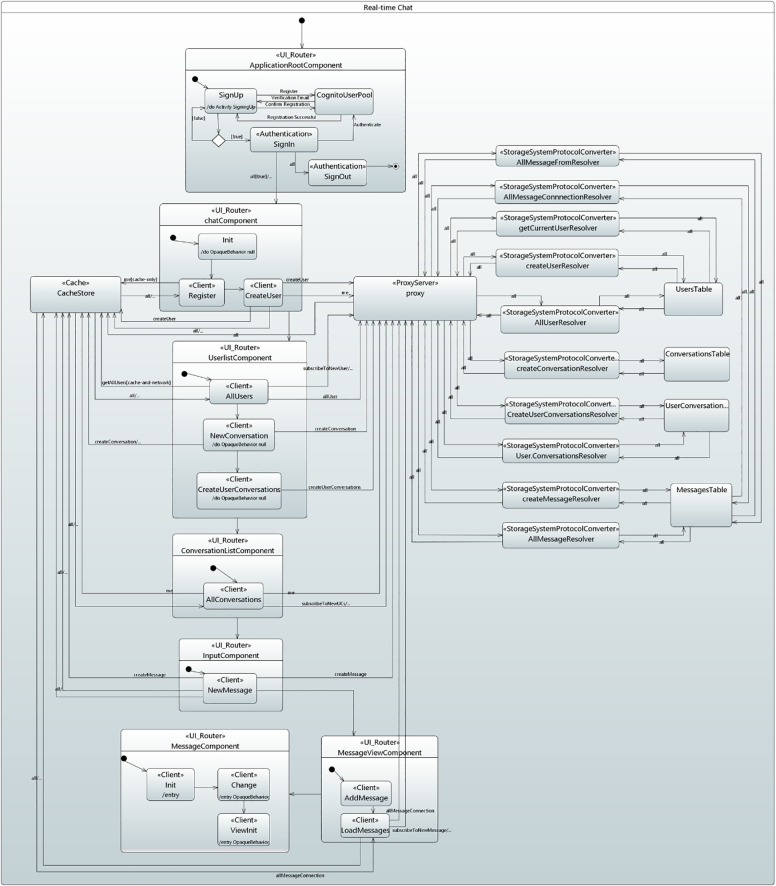
Behavioral model for the real-time chat application.

*Behavioral model of real-time chat application*. Fig 11 presents the behavioral model of Real-Time Chat Application in a state machine diagram.

The process starts by entering the application in ApplicationRootComponent state. The signUp state is activated to register the user through the cloud cognito user pool which verifies the user through a verification code. After successful verification, signIn state is activated and the Authentication stereotype is applied to perform user authentication. Upon successful authentication, a jwtToken is returned to the application that identifies the user and authorize access to the GraphQL API. The UI_Router stereotype is applied to route the application towards ChatComponent state. Authentication stereotype is applied to signOut state which is activated on exiting the application.

The ChatComponent state activates the “init” state which returns a cognito user session object, containing jwtToken. On entering the Register state, Client stereotype is applied to take the values for fetching the current logged in user from the cache. Similarly, the createUser state generates a user object with various data fields. Furthermore, Request_Data stereotype is applied to take the data fields for createUser request. Subsequently, the proxy receives and validates the request through the GraphQL API schema. The createUserResolver state adds the user data to the Amazon DynamoDB UsersTable through StorageSystemProtocolConverter stereotype. The response is returned, and the cache store is updated. The currentuser query request is sent to proxy which calls the getCurrentUserResolver to get the current user through StorageSystemProtocolConverter stereotype and ultimately updates the chat component user interface. Moreover, the data is supplied to the components through Input_Property and Output_Property parameters.

In the UserListComponent state, the Client stereotype is applied to AllUser state which defines the data fields and the corresponding operations, required to fetch the users from cache as well as network. Once the fields and operations are defined, Request_Data stereotype takes the data values for AllUser query request. The request is passed to proxy and the AllUserResolver fetches a list of all users from Amazon DynamoDB UsersTable through StorageSystemProtocolConverter stereotype. The client also opens a long-lived connection to the proxy by sending a subscription request. Once a new user is created in the Amazon DynamoDB Users Table, an event-based action is performed from the cloud through subscription that pushes the new user data to the user interface of all the subscribers connected to this application.

In NewConversation state, when the user invites another user to start a new conversation, Client stereotype define the data fields and operation for creating the new conversation. The GraphQL proxy validates the createConversation request with the GraphQL API schema and activates the createConversationResolver state. Subsequently, the cache store is updated with the returned data. Similarly, in createUserConversations state, createUserConversation request is created through Request_Data stereotype and all the corresponding actions are performed.

In ConversationListComponent, AllConversations state takes the data fields and operations through a Client stereotype to fetch the user conversations connection from the cache and network. Request_Data stereotype is applied to take the data fields and a proxy activates the userConversationsResolver state and returns the conversations connection to cache through StorageSystemProtocolConverter stereotype, which ultimately updates the conversation list component. From the UserConversation list, when the user wants to start a conversation, the ChatInputComponent state is activated. In this state, the user moves to NewMessage state and a new message is created through Client stereotype. Subsequently, Request_Data stereotype is mapped to create the data for createMessage request. In offline mode, an instant UI is created which is a response object with the message. The request is saved and automatically sent to the proxy when the network goes online. On network, the createMessageResolver state is activated that creates message with the above attributes in Amazon DynamoDB MessagesTable through the StorageSystemProtocolConverter stereotype.

The MessageViewComponent goes through the AddMessage and loadMessage states. The AddMessage state handles the retrieval of new messages through a Client stereotype. In LoadMessages state, simultaneous requests are made to get the corresponding conversation messages. The MessageViewComponent subscribes the data, if the messages are already in the cache. The transition is triggered on getconversationMessages, and therefore, Request_Data stereotype is applied to it. A getconversationMessages request is made with allMessageConnection query. The query returns a MessageConnection type, which is a GraphQL connection, consisting of data array and a token that indicates the starting point of the next query for the data so that the messages are retrieved in batches to maintain the application responsiveness. Upon entering the MessageComponent state, the init state reads the sender message through a query, defined through a Client stereotype. The change state handles the change everytime the message is updated and the ViewInit state handles the additional initialization tasks. Similarly, allMessageResolver and allMessageFromResolver states return the required message data values.

#### Transformation and deployment for real-time chat application

**Fig 12:**
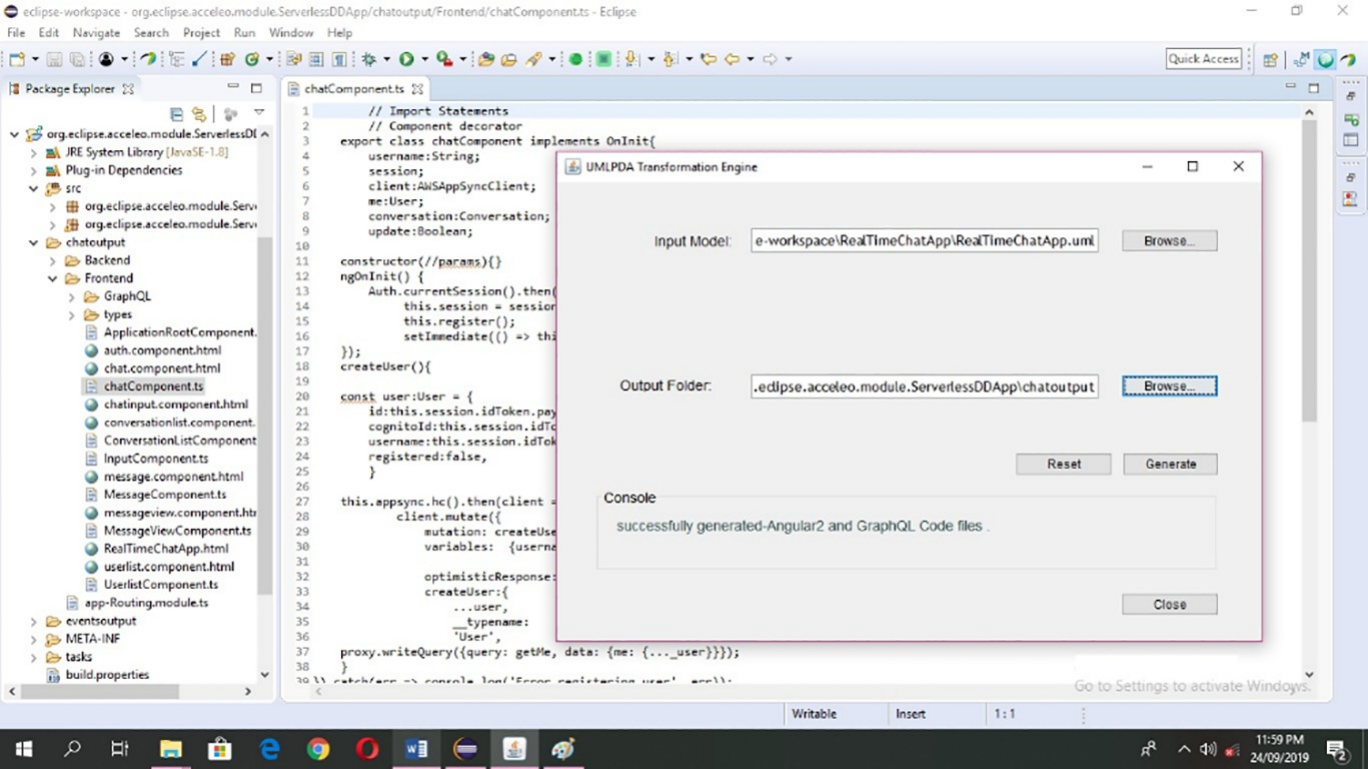
Transformation of code for real-time chat application.

UMLPDA transformation engine is used to automatically transform the Real-Time Chat Application model into the corresponding Angular 2 and GraphQL code, as shown in Fig 12.

**Fig 13:**
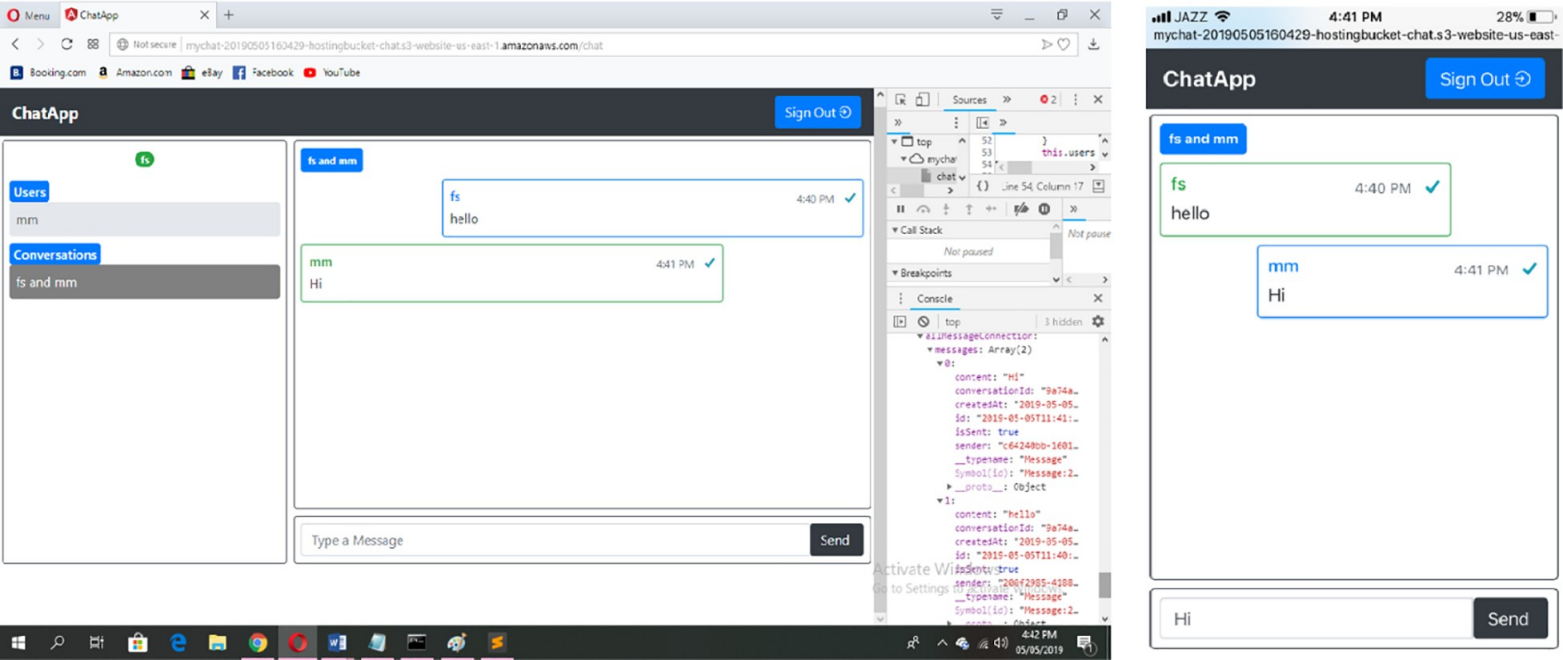
Real-time chat application after deployment.

The frontend code is deployed to Angular application and the backend GraphQL API (schema and resolver functions) is deployed to Amazon AWS AppSync serverless backend. The resources are created using AWS cloud formation template. Amazon DynamoDB [23] is used as a data source. The resources are allocated in response to a demand from the application. All requests are made to only one GraphQL endpoint. The cloud provider charges for the total number of operations and the real-time data updates to be performed by an application. Fig 13 shows the Real-Time Chat Application after deployment to AWS serverless platform.

### Events calendar application

We first discuss the requirements of Events Calendar Application. The corresponding modeling and implementation (transformation and validation) details are then provided.

#### Requirements for events calendar application

The main requirements of this case study are: (a) Authentication to secure and protect the data, (b) creating, viewing and deleting the events, (c) posting comments on existing events, (d) real-time updates to present comments to all the authenticated users.

#### Modeling of events calendar application

**Fig 14:**
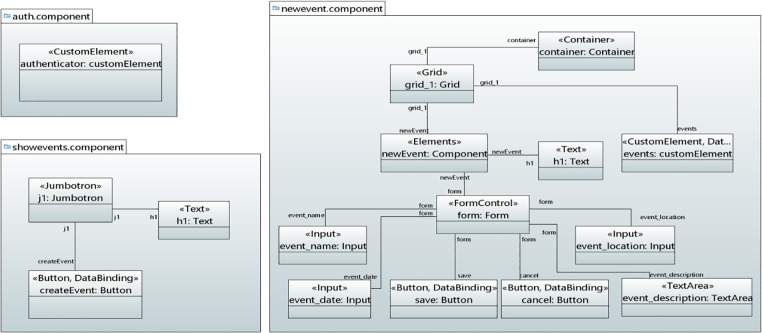
User interface for Auth, ShowEvents and NewEvent component.

**Fig 15:**
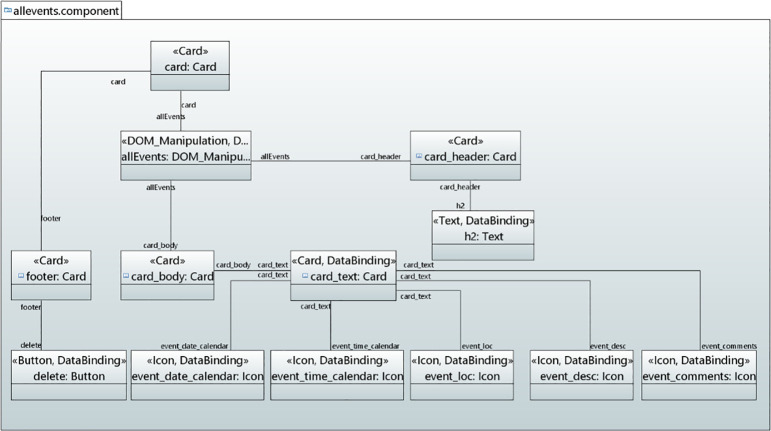
User interface for AllEvents component.

**Fig 16:**
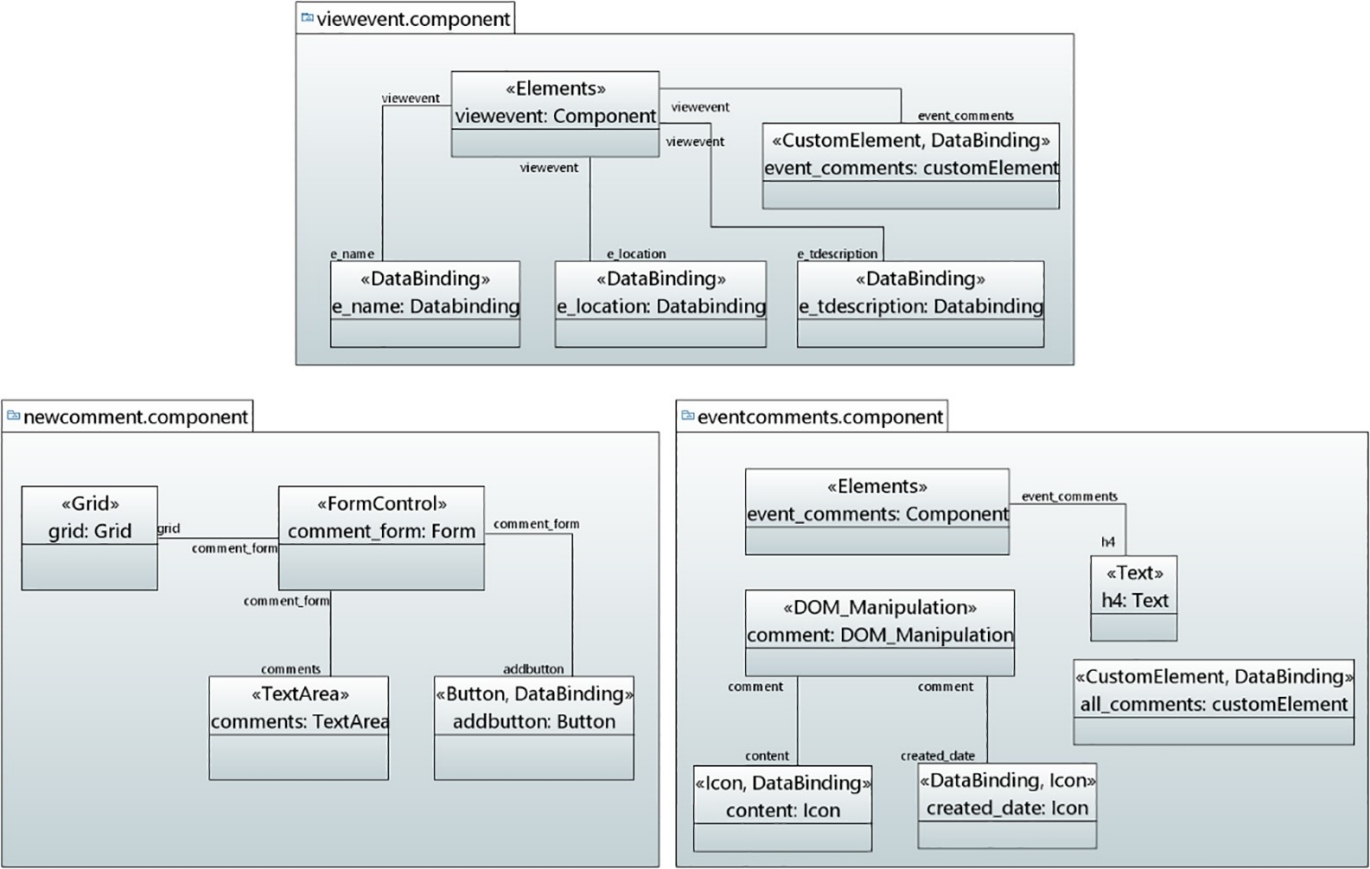
User interface for ViewEvent, NewComment and CommentOnEvent components.

*User interface model*. Figs 14–16 show the modeling of Events Calendar Application user interfaces in UML object diagram. Fig 14 shows the modeling of Auth, ShowEvents and New Event component. In Auth component, CustomElement stereotype is applied to add the user interface for authentication. In ShowEvents component, Jumbotron is applied to display the message. Text stereotype is applied for heading of the text while the Button stereotype is applied to button that takes the values to create an event button and DataBinding stereotype binds event to the button. In NewEvent component, Container, Grid, FormContol, Input, TextArea and Button stereotypes are applied to add the elements with layout and styling to the NewEvent form component.

Fig 15 shows the allevents component that displays all the events. In Fig 15, Card stereotype is applied that takes the values for card header, body, title and footer. Since card header contains heading, therefore, Text stereotype is mapped to take the values for card header. The data in the card is retrieved though DOM_Manipulation and DataBinding stereotypes. Icon stereotype is mapped to attach icon with the data or information.

Fig 16 shows the viewEvent, newComment and EventComment components. In the viewevent component, Elements stereotype is applied to add some styling to the component. DataBinding is applied to take the values for different binding types. In the newComment component, Grid, FormControl, TextArea and Button stereotypes are applied to take the values for the creation of a user interface. The eventComment Component displays the comments on the event by taking the values through Elements, Text, DOM_Manipulation, Icon and DataBinding stereotypes.

**Fig 17:**
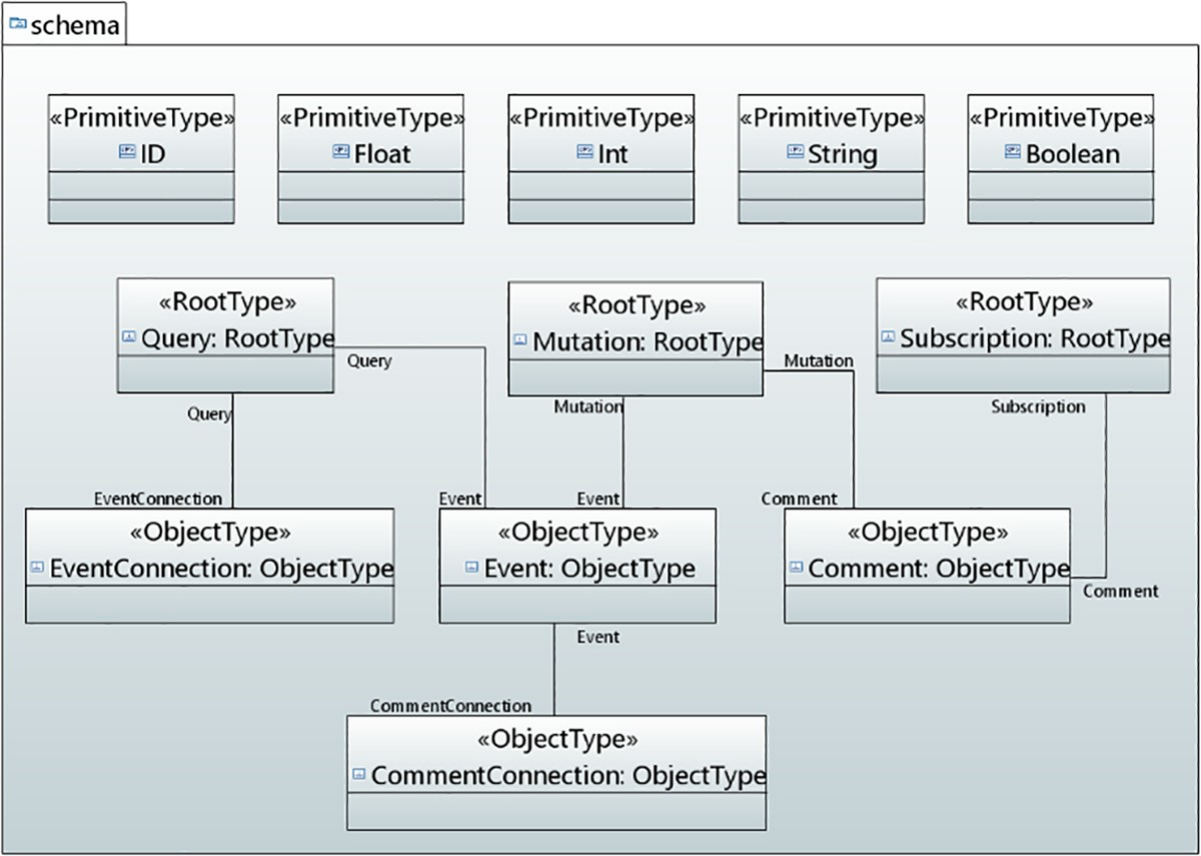
GraphQL API schema for events calendar application.

*Backend GraphQL API Schema Model*. Fig 17 shows the model for the backend API schema for Events Calendar Application. In this model, RootType stereotype is applied to define the Root Operations (Read, Write and Subscribe) for Events Calendar Application. The Operations in the RootType include createEvent, deleteEvent, commentOnEvent etc. ObjectType stereotype is applied to define the data fields for representing the data in the Event ObjectType. Similarly, other object types have their corresponding fields. The fields in the ObjectType and the parameters defined in the operation have some type modifiers. Consequently, a TypeModifier stereotype is applied to the fields and operation parameters.

**Fig 18:**
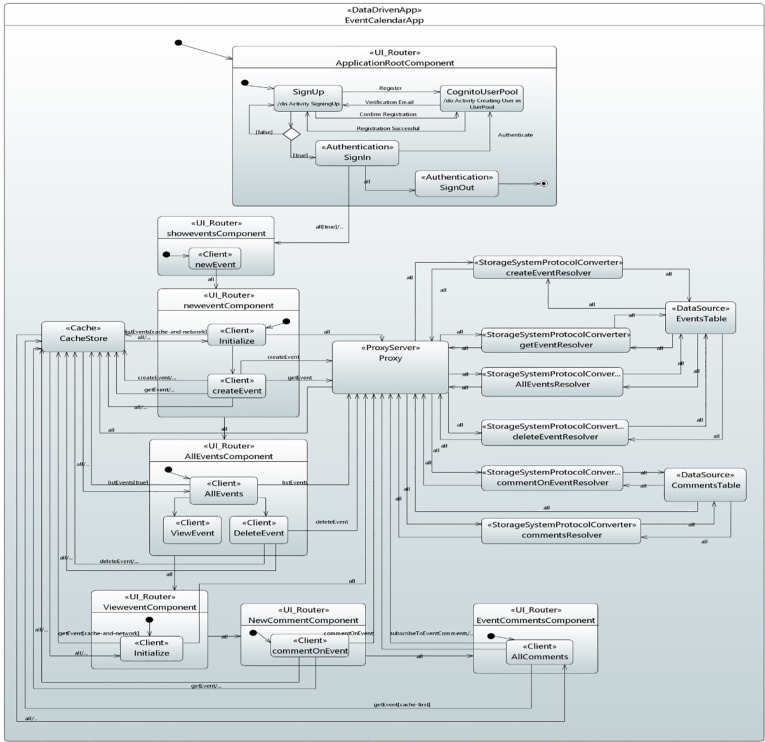
Behavioral model for event calendar application.

*Behavioral model for events calendar application*. The state machine diagram for the behavioral model of the Events Calendar Application is shown in Fig 18.

The process starts by entering the application in ApplicationRootComponent state. This state authenticates the users, as described previously in the real-time chat application case study. The application enters the showEventsComponent state through UI_Router stereotype after successful authentication. In the showEventsComponent state, the application moves to the newEvent state through which navigation to newEventComponent occurs. Upon entering the NewEventComponent state, the application moves to the initialize state, which fetches all the events from the cache as well as network through the Client stereotype. Subsequently, the proxy processes the received request and validate it with the GraphQL API schema. After validation, it activates the AllEventResolver state that gets all the events through StorageTransitionProtocolConverter stereotype by querying the Amazon DynamoDB Events Table. The response is returned, and the cache store is accordingly updated.

When the createEvent state is activated, the Client stereotype is mapped to construct an event object with various data fields. Then, the Request_Data stereotype is mapped to createEvent request to receive the corresponding data fields. Subsequently, the proxy validates the request with API schema and invokes the createEventResolver state to add the Event data to the Amazon DynamoDB EventsTable through StorageTransitionProtocolConverter stereotype. The response is returned, and the cache store is updated.

In AllEventsComponent state, the Client stereotype is applied to AllEvents state that takes the required data values for fetching all the events. The simultaneous query requests are made which returns an EventConnection type comprising of array of data as well as a token that indicates the starting point of next query for the data so that the events are received in batches. When the data is received, user can view the events by navigating to the viewEvent state. For deleting an event, user enters the deleteEvent state. Request_data stereotype takes the data fields for deleteEvent request. The request is sent to proxy along with the corresponding identity. The proxy processes the request and enters in the deleteEventResolver state to delete the event from the data source through StorageSystemProtocolConverter stereotype. Consequently, the corresponding event is deleted from the Amazon DynamoDB EventsTable.

In the viewEventComponent state, the Client stereotype is applied to initialize the state. If the cache contains no data, getEvent request is sent to the proxy, and therefore, getEventResolver state is activated to get the event from the Amazon DynamoDB EventsTable through StorageSystemProtocolConverter stereotype.

In NewCommentComponet state, the transition is triggered on commentOnEvent. Request_Data stereotype takes the data values for commentOnEvent request. The proxy activates the commentOnEventResolver state that adds comments to the CommentsTable using StorageTransitionProtocolConverter stereotype. Upon receiving the new comment in an Amazon DynamoDB CommentsTable, the new comment is pushed to an events application in real-time through subscription and the cache store is updated.

In the EventCommentsComponent state, the Client stereotype is mapped to getAllComments state that takes the values for getting all the comments. The transition is triggered on getEvent, created through Request_Data stereotype. The request is passed to the cache to fetch all the comments. This state subscribes the new comment, returned from the network through subscribeToEventComments subscription. Similarly, commentsResolver state returns all the comments belonging to an individual event.

#### Transformation and deployment

**Fig 19:**
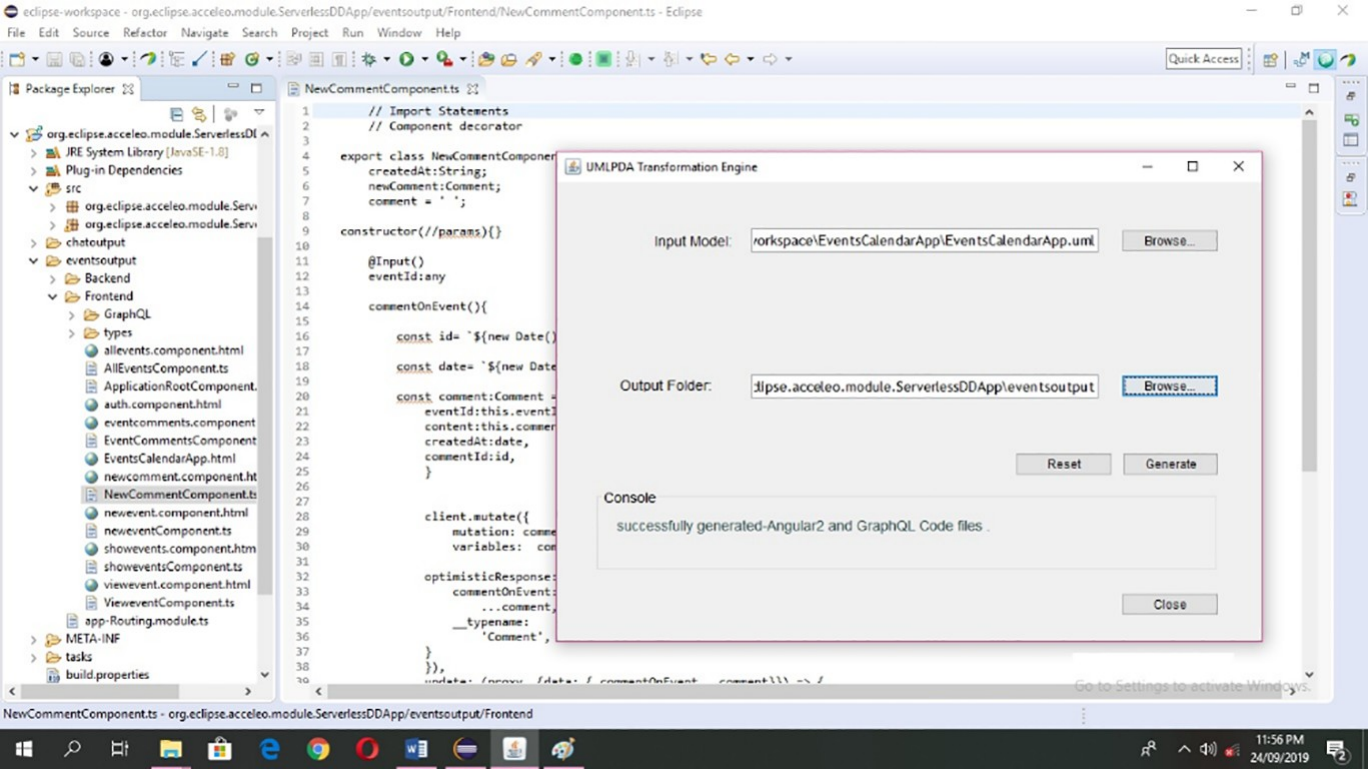
Transformation of code for events calendar application.

Fig 19 shows the Events Calendar Application code generation through UMLPDA transformation engine.

**Fig 20:**
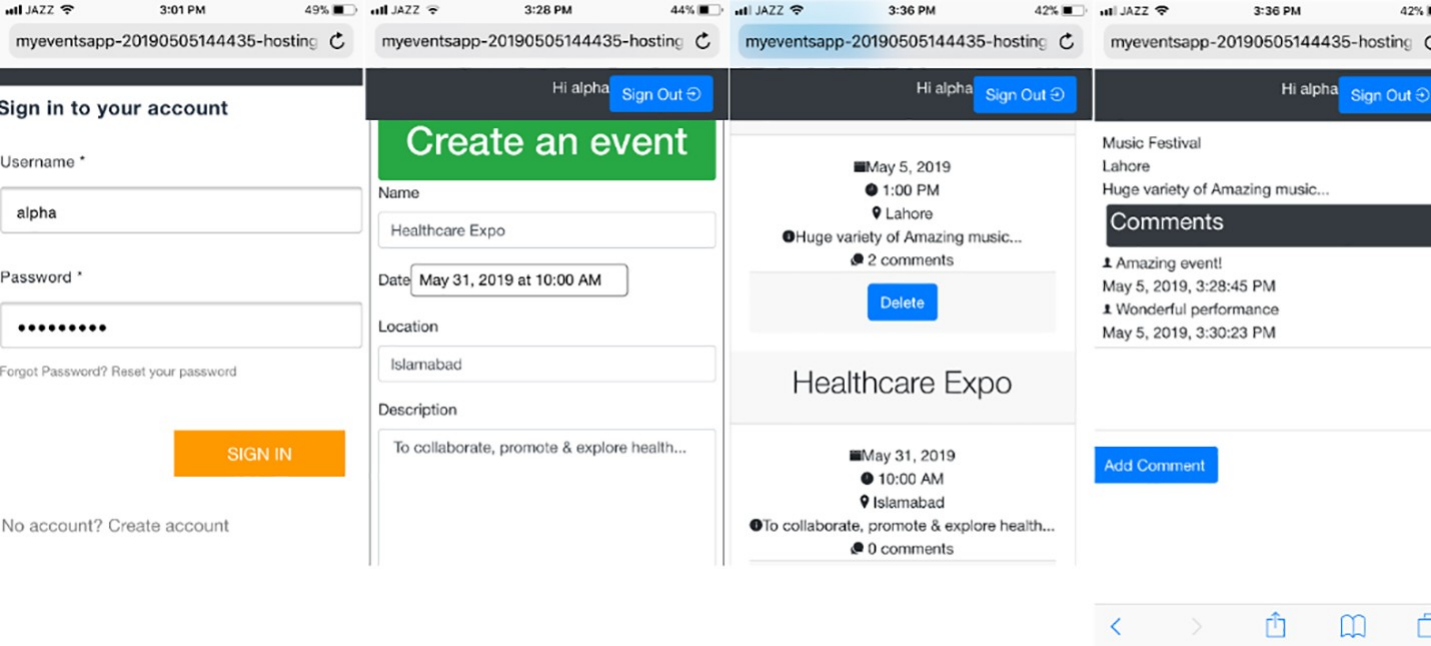
Events calendar application after deployment.

Fig 20 shows the Events Calendar Application after deployment.

### Movies application

This case study is used to add the movie specific information. It also supports the real-time updates for presenting users with the updated information. It supports the following key requirements: (a) authentication and authorization, (b) add, view, update and delete the movies, (c) a like feature to indicate the response of the audience, (d) add reviews and ratings to the movie and (e) the real-time updates for new movie and movie reviews. We do not include the modeling and code generation details of Movies Application due to the space limitations. The details of this application are available online at [49].

This section has demonstrated the applicability of UMLPDA through three case studies. An open source transformation engine has been developed and used to generate an accurate Angular2 and GraphQL code for all the three case studies from the given models. The source code for transformation engine along with the corresponding case studies can be found at [49]. Although, the UMLPDA generates code for Angular2 and GraphQL, however, there exist some limitations. It is not possible to incorporate all the features in a single research. The frontend of an application does not cover all the user interface elements. Also, it does not completely provide full styling of UI elements. Furthermore, the transformation rules for other types in the backend GraphQL API schema such as Input, Union, Interface and ComplexObject types have not been provided yet. Moreover, some manual input is required for adding the import statements as per the desired function.

### Discussion on the effectiveness of proposed approach

To this point, we have demonstrated the applicability of proposed work (UMLPDA profile and its transformation engine) with three different case studies. This section will present the benefits of the proposed approach by discussing the efficiency of UMLPDA with respect to low level implementation of data-driven applications in terms of required development efforts. It will further strengthen our claim that the proposed approach simplifies the development of data-driven applications in a serverless cloud computing environment.

In order to evaluate the effectiveness of our approach, two students (student A and student B) from Software Engineering department with appropriate programming background were selected. Both students provided informed consent to participate in this study. Before actually assigning the development task for data-driven applications, both students have undergone a training phase. During the training period, student A learned the concepts of UMLPDA profile while student B acquired the concepts of data-driven applications including user interface, behavior and GraphQL API concepts. Additionally, both students also learned the serverless deployment concepts of an application. After the training period, student A was assigned to develop a data-driven application through the proposed profile while student B was asked to develop the same data-driven application in a serverless cloud computing framework with conventional low-level implementation. During the development process, student A created UML models for the assigned case study and applied the UMLPDA concepts. Subsequently, he generated the low-level implementation code automatically through the proposed transformation engine. On the other hand, student B developed the same case study through the conventional programming approach at lower level of implementation.

It has been observed that student A generated the target code through the proposed profile and its transformation engine with simplicity. However, some extra time is required for deploying code in its respective environment and then tracing the code for manual fixing, such as adding import statements and writing serverless deployment code, to make a full working application. By summing up all, student A has taken 90 hours for the development of a complete case study (including the training time). At the same time, student B has taken 200 hours for the development of same case study through conventional programming style (low level implementation). In other words, the proposed profile has reduced the development efforts significantly as compared to low level implementation.

## Conclusions

This article presents a novel model-driven approach to simplify the data-driven applications development in serverless cloud computing. Particularly, the UMLPDA has been proposed which adopts the concepts of UML-based Model-driven Architectures to model the design and implementation of both frontend and backend for data-driven applications in serverless cloud computing at a higher abstraction level. The proposed UMLPDA consists of several stereotypes, related to the frontend and backend concepts. Subsequently, a complete open source transformation engine is developed to transform the UMLPDA source models into the target low-level implementation of Angular2 and GraphQL code. The transformation engine implementation is carried out in Acceleo through a Model to Text approach. We demonstrate the applicability of proposed framework/tool through three case studies, deployed on AWS Serverless platform. The results reveal that the proposed framework allows the modeling of both frontend as well as backend requirements of data-driven applications in serverless cloud computing with simplicity and the corresponding transformation engine automatically generates the target code with high accuracy.
